# Adipocytes promote breast cancer resistance to chemotherapy, a process amplified by obesity: role of the major vault protein (MVP)

**DOI:** 10.1186/s13058-018-1088-6

**Published:** 2019-01-17

**Authors:** Camille Lehuédé, Xia Li, Stéphanie Dauvillier, Charlotte Vaysse, Camille Franchet, Emily Clement, David Esteve, Mélanie Longué, Léonor Chaltiel, Sophie Le Gonidec, Ikrame Lazar, Aline Geneste, Charles Dumontet, Philippe Valet, Laurence Nieto, Frédérique Fallone, Catherine Muller

**Affiliations:** 1 Institut de Pharmacologie et de Biologie Structurale (IPBS), Université de Toulouse, CNRS, UPS, UMR 5089, 205 route de Narbonne, 31077 Toulouse, France; 2 Institut des Maladies Métaboliques et Cardiovasculaires (I2MC), Université de Toulouse, INSERM, UPS, Toulouse, France; 3 Département de Chirurgie Oncologique, Centre Hospitalier Universitaire de Toulouse, Institut Universitaire du Cancer de Toulouse-Oncopole, Toulouse, France; 4 Service d’Anatomo-Pathologie, Centre Hospitalier Universitaire de Toulouse, Institut Universitaire du Cancer de Toulouse-Oncopole, Toulouse, France; 5 Département de Biostatistiques, Institut Claudius Regaud, Institut Universitaire du Cancer de Toulouse-Oncopole, Toulouse, France; 6 Centre de Recherche en Cancérologie de Lyon (CRCL), INSERM UMR 1052/CNRS, Lyon, France; 7 Present address: Department of Oncology, The First Affiliated Hospital of Chongqing Medical University, Chongqing, China

**Keywords:** Adipocytes, Breast cancer, Obesity, Doxorubicin, Multidrug resistance, Mammary adipose tissue

## Abstract

**Introduction:**

Clinical studies suggest that obesity, in addition to promoting breast cancer aggressiveness, is associated with a decrease in chemotherapy efficacy, although the mechanisms involved remain elusive. As chemotherapy is one of the main treatments for aggressive or metastatic breast cancer, we investigated whether adipocytes can mediate resistance to doxorubicin (DOX), one of the main drugs used to treat breast cancer, and the mechanisms associated.

**Methods:**

We used a coculture system to grow breast cancer cells with *in vitro* differentiated adipocytes as well as primary mammary adipocytes isolated from lean and obese patients. Drug cellular accumulation, distribution, and efflux were studied by immunofluorescence, flow cytometry, and analysis of extracellular vesicles. Results were validated by immunohistochemistry in a series of lean and obese patients with cancer.

**Results:**

Adipocytes differentiated *in vitro* promote DOX resistance (with cross-resistance to paclitaxel and 5-fluorouracil) in a large panel of human and murine breast cancer cell lines independently of their subtype. Subcellular distribution of DOX was altered in cocultivated cells with decreased nuclear accumulation of the drug associated with a localized accumulation in cytoplasmic vesicles, which then are expelled into the extracellular medium. The transport-associated major vault protein (MVP), whose expression was upregulated by adipocytes, mediated both processes. Coculture with human mammary adipocytes also induced chemoresistance in breast cancer cells (as well as the related MVP-induced DOX efflux) and their effect was amplified by obesity. Finally, in a series of human breast tumors, we observed a gradient of MVP expression, which was higher at the invasive front, where tumor cells are at close proximity to adipocytes, than in the tumor center, highlighting the clinical relevance of our results. High expression of MVP in these tumor cells is of particular interest since they are more likely to disseminate to give rise to chemoresistant metastases.

**Conclusions:**

Collectively, our study shows that adipocytes induce an MVP-related multidrug-resistant phenotype in breast cancer cells, which could contribute to obesity-related chemoresistance.

**Electronic supplementary material:**

The online version of this article (10.1186/s13058-018-1088-6) contains supplementary material, which is available to authorized users.

## Background

Studying the multifaceted role of adipocytes in cancer, and particularly in breast cancer, is of major clinical importance. Indeed, obesity, where the normal balance of adipose tissue (AT) secretions is perturbed, is a negative prognosis factor for breast cancer independently of menopausal status (for review, see [1, 2]). There is now clear evidence that, in obese women, host factors contribute to the occurrence of tumors exhibiting an aggressive phenotype at diagnosis, defined by an increase in lymph node involvement and a higher propensity to distant metastasis (for review, see [1]). AT represents a major component of the breast tumor microenvironment, and numerous studies now indicate that the mammary AT (MAT) adjacent to tumors supports breast cancer development and progression [2]. We and others have demonstrated that a bidirectional crosstalk takes place between breast cancer cells and tumor-surrounding AT [3–6]. Tumor-surrounding adipocytes stimulate cancer invasiveness by secreting extracellular matrix and matrix metalloproteases, pro-inflammatory cytokines, and modulation of cancer cell metabolism, processes likely to be amplified in obese patients [3–6]. In addition to increased tumor aggressiveness, the poor prognosis observed in obese patients is related to decreased response to treatment, an aspect of the adipocyte/tumor crosstalk that is probably underestimated [7]. At a clinical level, several studies provide evidence that adjuvant and neo-adjuvant chemotherapies are less effective in obese women [8–10]. However, limited data exist at a laboratory level to demonstrate a paracrine role of adjacent MAT in breast cancer drug resistance. One study shows that endotrophin, a cleavage product of collagen VI alpha 3 chain, causes resistance to cisplatin [11]. In other cancers, several studies highlight that adipocytes promote drug resistance with modulation of cell death pathways as the main responsible mechanism [7]. We have demonstrated that, besides chemotherapy, adipocytes contribute to the occurrence of a radioresistant phenotype in breast tumor cells through increased activation of the effector kinase Chk1 [12]. Adipocytes also inhibit trastuzumab-mediated antibody-dependent cellular cytotoxicity in HER2-expressing breast cancer cells via the secretion of soluble factors [13].

Evaluating the role of MAT in resistance to chemotherapy is of major clinical importance since this approach remains the basic treatment for aggressive or metastatic breast cancers. In accordance with its multiple mechanisms of action, leading to DNA damage and tumor cell death, doxorubicin (DOX), which belongs to the anthracycline family, is considered one of the most effective agents for breast cancer treatment [14]. Despite extensive clinical use, intrinsic or acquired resistance to this drug is frequent and represents one of the major obstacles in breast cancer treatment. DOX-resistant cancer cells frequently exhibit cross-resistance to multiple unrelated classes of anti-cancer drugs. This mechanism, known as multidrug resistance (MDR), is a major cause of therapeutic failure [15]. One of most predominant processes underlying MDR is increased drug efflux, leading to a decrease in drug accumulation at intracellular targets. The role of the ABC (ATP-binding protein) transporter family in this process has been extensively studied. These proteins can expel several substrates from cells in an ATP-dependent manner against a drug concentration gradient [15]. Among all human ABC transporters, three are known to be mainly involved in DOX efflux: ABCB1 (Pgp for P-glycoprotein), ABCC1 (MRP1 for multidrug resistance-associated protein 1), and ABCG2 (BCRP for breast cancer resistance protein) [15]. Overexpression of another actor, LRP (lung resistance protein), has been found in a number of tumor cell lines that exhibit an MDR phenotype, especially P-gp–negative lines [16, 17]. Subsequently, LRP was identified as the human major vault protein (MVP), which is the main component of vault particles [18, 19]. These large particles exhibit a hollow barrel-like structure, are localized mainly in the cytoplasm, and are associated with the nucleus [20, 21]. Vault particles are involved in intracellular transport of molecules, including anti-cancer drugs (for review, see [21]). Despite some controversy, overexpression of MVP has been correlated with the degree of malignancy and drug resistance, resulting in a poor prognosis for cancer treatment with several drugs, including DOX [18, 21]. In the present study, we demonstrate for the first time that tumor-surrounding adipocytes promote resistance to DOX and other unrelated chemotherapeutic agents through an MVP-dependent mechanism, a process that is increased by obesity. In human breast cancer, MVP is upregulated in tumor cells at the invasive front, where they are in close contact to adipocytes, highlighting the clinical relevance of our results. This study reveals a new role of MAT in promoting breast cancer aggressiveness and could provide interesting opportunities to set up specific strategies for the treatment of obese patients exhibiting aggressive breast cancer.

## Methods

### Reagents

Mouse monoclonal antibody (mAb) against MVP (clone 42/LRP), used for western blot and immunohistochemistry, was purchased from BD Biosciences (San Jose, CA, USA). The mAb directed against α tubulin (clone DM1A) is from NeoMarkers (Fremont, CA, USA). DOX, paclitaxel, and 5-fluorouracil (5-FU) were from Sigma-Aldrich (St. Louis, MO, USA). Mafosfamide, a stable active analog of 4-OH-cyclophosphamide, was from Santa Cruz Biotechnology (Dallas, TX, USA). The P-gp inhibitors verapamil (used at 40 μM) and tariquidar (used at 75 nM) were respectively from Sigma-Aldrich and Selleckchem (Houston, TX, USA). The MRP family inhibitor MK-571 (MK) and the BCRP inhibitor Fumitremorgin-C, used at 30 μM, were from Sigma-Aldrich. All the reagents were diluted in dimethyl sulfoxide (except DOX, diluted in water) and used within the efficient pharmacological range of doses described in the literature [22–25]. BODIPY™ 493/503 (used at 1 μg/mL) and 4′,6-diamidino-2-phenylindole (DAPI) (1 μM) were from Life Technologies (Grand Island, NY, USA). Hoechst 33342 was from Invitrogen (Auckland, New Zealand).

### Cell lines and culture

Breast cancer cell lines were cultivated as follows: T47D and MDA-MB231 (provided by K. Bystricky, LBME, Toulouse, France) as well as MDA-MB453 and BT-474 cells (provided by C. Dumontet, CRCL, Lyon, France) were grown in RPMI 1640 medium. MDA-MB436 cells (provided by I. Martin Padura, IEO, Milan, Italy) were cultured in Dulbecco’s modified Eagle’s medium/F-12 (DMEM/F-12) (1:1). The murine breast cancer cell lines M-Wnt [26] (provided by S. Hursting, Chapel Hill, NC, USA) and E0771 [27] (provided by J. Nunes, CRCM, Marseille, France) were cultured in RPMI 1640 medium (supplemented with 10 mM HEPES pH 7.5 for the E0771 cell line). All the culture reagents were from Invitrogen. The murine 3 T3-F442A preadipocyte cell line (obtained from the European Collection of Cell Cultures, Public Health England, UK) was grown in DMEM and differentiated into mature adipocytes as previously described [3]. The term “adipocyte” refers to cells that have been differentiated for 10 to 14 days (with 80% of cells accumulating lipid droplets). Culture media were supplemented with 10% fetal calf serum (FCS) and antibiotics (125 mg/mL streptomycin and 125 UI/mL penicillin). All cells were grown in a humid atmosphere with 5% CO_2_. All cell lines were used within 2 months after thawing of frozen aliquots and were authenticated on the basis of viability, recovery, growth, and morphology. The cells were tested every month by polymerase chain reaction for mycoplasma contamination.

### Coculture system, preparation of adipocyte-conditioned medium, and isolation of soluble factors

Tumor cells and adipocytes were cocultivated by using a Transwell culture system (0.4-μm pore size; Millipore, Burlington, MA, USA) as previously described [3]. Murine or human breast tumor cells were seeded in the upper chamber of the Transwell system in the same culture medium as adipocytes and were cocultivated (or not) with 3 T3-F442A adipocytes for the indicated times (3 to 6 days). Adipocyte-conditioned medium (AdCM) was obtained from 3 T3-F442A adipocytes as previously described [3]. For the isolation of adipocyte soluble factors (SF), 3T3-F442A adipocytes were cultivated for 48 h with complete DMEM without phenol red (to avoid any potential fluorescence contamination) previously depleted of contaminating bovine vesicles by overnight centrifugation at 100,000*g*. This medium was then collected and centrifuged at 3000*g* for 30 min and at 10,000*g* 60 min and, finally, ultracentrifuged overnight at 100,000*g*.

### Isolation of human mammary adipocytes and three-dimensional culture

Human MAT samples were collected from mastectomies at Institut Universitaire du Cancer de Toulouse (France) in accordance with ethics approval by our institutional review board. All patients gave their informed consent to participate in biological studies. MAT samples were collected at a distance from tumors and were macroscopically devoid of fibrosis. Samples were obtained from normal weight (NW) or obese women (mean body mass index (BMI) 22.4 ± 1.3 kg/m^2^ and 31.5 ± 1.5 kg/m^2^, respectively). Samples were collected and quickly processed after surgery. Adipocytes were separated from the stromal vascular fraction as previously described [3]. To perform coculture experiments, isolated adipocytes (300 μL) were embedded in a three-dimensional (3D) fibrin matrix composed of fibrinogen (6 mg/mL; 600 μL) and thrombin (12 U/mL; 900 μL) (both obtained from Sigma-Aldrich) in a Transwell culture system (0.4-μm pore size; Millipore) placed in a six well plate. The culture dish was immediately placed at 37 °C for 10 min to allow the gel to clot before adding adipocyte culture medium. For coculture, indicated breast cancer cells were seeded in the bottom compartment of the plates. For control conditions, tumor cells were placed with Transwells containing 3D fibrin matrix without adipocytes in order to take into account any factors released by the matrix that could affect tumor cell behavior.

### Drug treatment and viability assays

Breast tumor cells were cultured (or not) with adipocytes for 3 days with 3T3-F442A adipocytes or 2 days with human mammary adipocytes (pre-incubation period). Tumor cells then were treated alone with drugs for 4 h and washed once with fresh medium before being placed back with adipocytes (or not) for 3 days (post-incubation period). To measure viability, the Transwell chambers containing tumor cells were fixed with methanol, incubated with Toluidine Blue 1% in 0.1 M borax, and then washed abundantly. It has previously been shown that Toluidine Blue could efficiently stain fixed cells to provide the basis for a quantitative assay of cell number and drug cytotoxicity [28]. For analysis, treated cells were normalized to untreated cells. Of note, we have previously shown that coculture of breast cancer cells with adipocytes does not influence cell proliferation [3, 6]. The Transwell chamber membranes then were placed in a lysis buffer (6.25 mM Tris-HCl pH 6.8, 10% glycerol, 2% SDS, 5% β-mercaptoethanol), and the absorbance was measured at 570 nm (MicroQuant; BioTek Instrument Inc., Winooski, VT, USA). For experiments using RNA interference, viability was assessed by using an IncuCyte ZOOM Live-Cell Imaging system (Essen Bioscience, Ann Arbor, MI, USA). E0771 cells were seeded into 96-well plates (2 × 10^4^ cells per well) in complete DMEM and incubated overnight before replacement with fresh medium containing DOX for 4 h. After treatment, fresh DMEM or AdCM was added and plates were transferred to the IncuCyte for confluence analysis. Data shown are from four replicates (two images per time point). For time-lapse experiments, MDA-MB436 cells were seeded in Lab-Tek chambers (Thermo Fisher Scientific, Waltham, MA, USA) (3.5 × 10^4^ cells per well) in complete DMEM and incubated overnight before replacement with fresh medium containing DOX (3 μg/mL) for 4 h. After 4 h, fresh DMEM or AdCM was added and cells were analyzed at the indicated time points. Just before microscopy analysis, nuclei were counterstained using Hoechst 33342 (used at 1.6 μM for 12 min).

### DOX intranuclear and intracellular accumulation analysis

As DOX is inherently fluorescent, its presence within cells can be directly analyzed by the following approaches. For measure of DOX intranuclear accumulation, tumor cells grown on glass coverslips cocultivated (or not) with adipocytes were treated with DOX following the protocol described above. At indicated times, cells were fixed with 3.7% paraformaldehyde (PFA) for 20 min at room temperature. PFA was quenched with 50 mM NH_4_Cl and cells were permeabilized with 0.2% TritonX-100 for 5 min and then stained with DAPI for 5 min. After two washings with phosphate-buffered saline (PBS), slides were mounted in Vectashield medium (Vector Laboratories, Inc., Burlingame, CA, USA). Fluorescence images were acquired with a confocal laser microscopy system with a 60X oil PLAPON OSC objective (Confocal TIRF Olympus FV1000; Olympus, Tokyo, Japan). The intensity of staining in the nuclei was quantified on original images by using Fiji software. Images were processed to filter the noise with Fiji software (Image J, Bethesda, MD, USA) and a similar filter was used to analyze all acquisitions. To measure intracellular DOX accumulation by flow cytometry, in indicated experiments, cocultivated and non-cocultivated cells were treated (or not) with ABC transporter inhibitors, 1 h before DOX treatment and until the end of the experiment. At the end of the incubation, cells were washed with cold PBS, pelleted by centrifugation at 4 °C, and analyzed with a FACScalibur cytometer (Becton Dickinson, Rutherford, NJ, USA) using a 488-nm excitation and 670-nm long-pass filter (FL3-H). Mean fluorescence intensity was quantified by CellQuest software (Becton Dickinson). For each analysis, 20,000 events were collected.

### Western blot analysis

To prepare protein extracts, cells were trypsinized and pelleted by centrifugation at 4 °C and then placed on ice for 20 min in a lysis buffer containing 10 mM Tris-HCl pH 7.5, 150 mM NaCl, 5 mM EDTA, and 0.5% Triton X-100 with protease and phosphatase inhibitors (Sigma-Aldrich). After centrifugation (16,000*g*, 20 min, 4 °C), the supernatant was collected and stored at −20 °C. Western blots were performed as previously described [3].

### Small interfering RNA-mediated knockdown

Transient transfection of E0771 breast tumor cells with small interfering RNAs (siRNAs) was performed with Lipofectamine^®^ RNAiMAX Reagent (Life Technologies) in accordance with the instructions of the manufacturer. In brief, transfection was performed in six well plates containing 50% confluent cells in appropriate medium to which the transfection mix was added to a final siRNA concentration of 100 nM. Five hours after transfection, medium was replaced with fresh DMEM containing 10% FCS. The three siRNAs directed against murine MVP used were from Qiagen (Hilden, Germany). Their target sequences are as follows: siMVP1 (also named siMVP), 5′-CUGGCACUUUGAACUGAAGAA-3′; siMVP2, 5′-TACACTGACAATGGATAAATA-3′; and siMVP3, 5′-CTGGACTTTGAAGATAAGAAT-3′. Control siRNA containing an untargeted sequence was provided by Qiagen (SI1022076). For experiments using drugs (DOX, 5-FU), cells were treated 24 h after the transfection. After treatment, cells were analyzed immediately (0 h) or (i) cocultivated (or not) with adipocytes and (ii) incubated (or not) with AdCM for the indicated times.

### Tumor cell extracellular vesicles preparation and quantification

Tumor cells were pre-incubated (or not) with adipocytes in medium without phenol red and then treated alone with DOX for 4 h. After DOX removal, tumor cells were incubated (or not) for 18 h with adipocyte soluble factors (SF) obtained from AdCM previously depleted of bovine vesicles. This SF fraction depleted of vesicles was used rather than coculture with adipocytes or AdCM in order to obtain only tumor-derived extracellular vesicles (EVs) at the end of the experiment. The tumor cell–conditioned medium then was collected and centrifuged (30 min, 3000*g*) to remove cell debris contamination. All steps were carried out at 4 °C. The concentration and size distribution of EVs were analyzed with a NanoSight LM10-HS (Malvern, Orsay, France) equipped with a 405-nm laser as previously described [29]. In certain experiments, EVs were conjugated to 4 μm–diameter aldehyde/sulfate latex beads (Life Technologies) in order to measure DOX content by flow cytometry. To this end, 25 × 10^8^ vesicles were incubated with 150,000 beads in PBS for 15 min under gentle agitation at room temperature followed by overnight incubation at 4 °C. Note that the optimal bead-to-vesicle ratio was chosen after preliminary experiments. The vesicle-coated beads were analyzed with a FACScalibur cytometer. Mean fluorescence intensity was quantified by CellQuest software (Becton Dickinson). For each analysis, 20,000 events were collected.

### MVP staining and quantification

Immunohistochemical staining for MVP was performed in 34 whole slices of human breast tumors (see Additional file 1: Supplementary material and methods and Additional file 2: Table S1 for full clinicopathological characteristics of the patients included in this pilot study) using anti-MVP antibody (1:400) with associated hematoxylin and eosin (HE) slides for morphological control. Validation of the antibody used was performed with appropriate positive and negative controls in accordance with Human Protein Atlas and literature [30–32] (Additional file 3: Figure S1). The immunostaining was performed on an AutoStainer Link 48 (Dako, Glostrup, Denmark). We decided to choose tumors surrounded by fat tissue, and we excluded tumors if thick connective tissue was present between the tumor and fat tissue. One breast pathology expert and one trained surgeon, blind to the clinical data, independently scored MVP expression in human tumors as negative, low, moderate, or high. The slides were then digitally scanned with a Hamamatsu Nanozoomer 2.0RS (Hamamatsu Photonics, Shizuoka, Japan) and analyzed with the device software provided by the manufacturer. To quantify the MVP immunostaining intensity for each tumor, five areas at the tumor invasive front and five areas in the tumor center were first manually selected on HE slides by the pathologist and then reported on the corresponding MVP slides. The intensities of MVP staining were quantified by using Fiji software, and tumor staining was separated by using a deconvolution plug-in. First, we used a color deconvolution technique to separate the MVP signal from the aspecific signal, leaving a complementary image. The integrated intensities of MVP labelling images (positive areas for MVP × mean of intensity) were then determined. Finally, we ensured that the results obtained were in accordance with the four groups previously defined by manual scoring. Descriptive characteristics are presented by median (range) for continuous variables and by frequency (percentage) for categorical variables. As BMI (kg/m^2^) is the most frequent anthropometric tool used in clinical practice, the cohort was split into two groups: normal weight (BMI <25 kg/m^2^) and overweight/obese (BMI ≥25 kg/m^2^).

### Statistical analysis

Comparisons between groups were performed by using Mann–Whitney *U* test. The Benjamini–Hochberg procedure was applied for multiple comparisons. All reported *P* values were two-sided. Statistical analysis was performed by using R 3.2.2 software. Bar and errors flags represent mean ± standard error of the mean of at least three independent experiments. For all statistical tests, differences were considered significant at the 5% level (*P values <0.05, **P values <0.01, ***P values <0.001, and ****P values <0.0001).

## Results

### Coculture with mature adipocytes promotes a multidrug-resistance phenotype in a wide panel of human and murine breast cancer cell lines

**Fig. 1:**
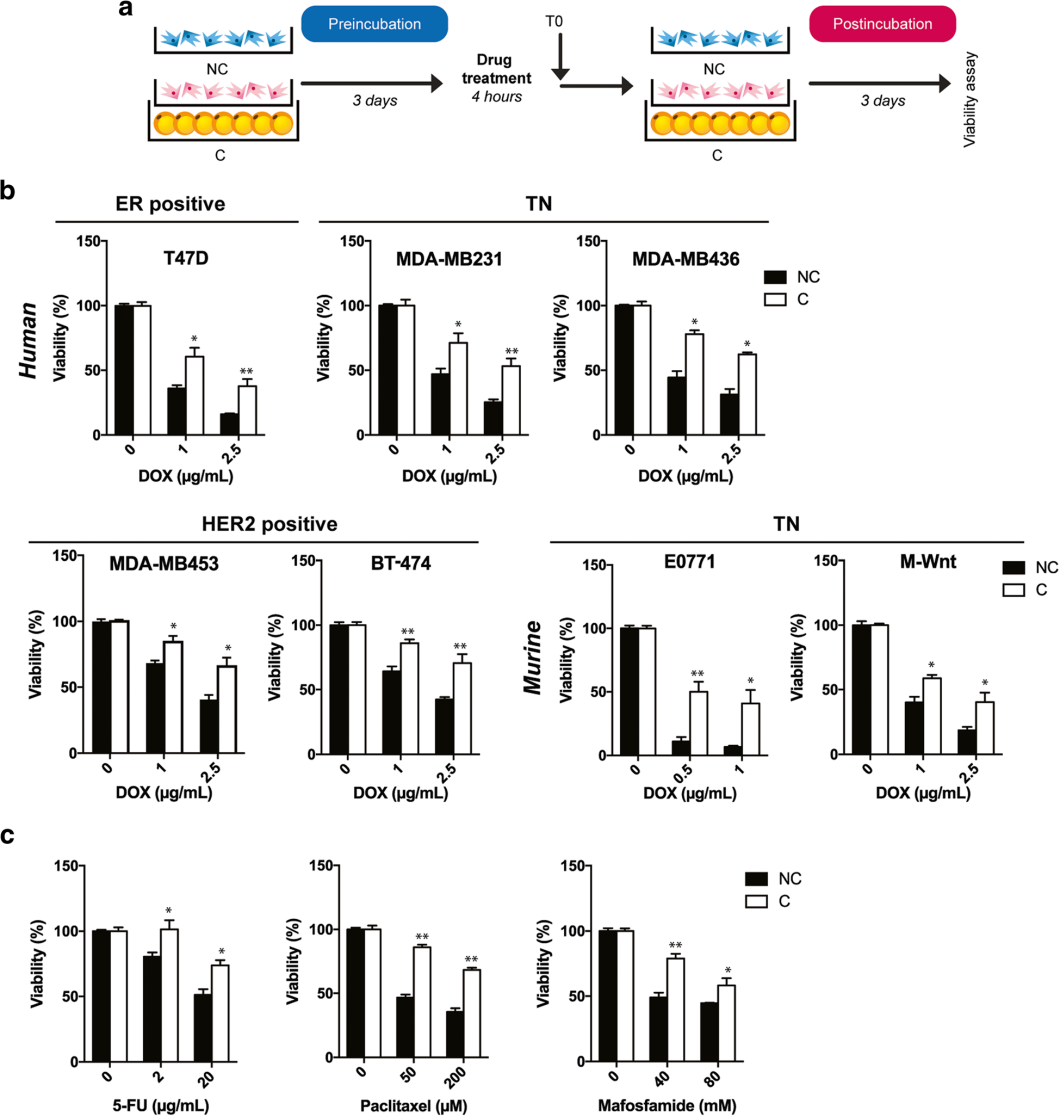
Coculture with adipocytes promotes multidrug resistance in various human and murine breast cancer cell lines. **a** Experimental design: breast tumor cells were cocultivated (pink cells) (or not) (blue cells) with adipocytes for 3 days (pre-incubation period) and incubated alone with drugs for 4 h. After washing, cells were cultivated again (or not) with adipocytes for 3 days (post-incubation period). At the end of the post-incubation period, the number of viable cells was determined. **b** Results of viability assays with various breast tumor cells cocultivated (white bar) (or not) (black bar) with adipocytes and treated (or not) with doxorubicin (DOX) at indicated doses. Bar plots represent the percentage of surviving cells relative to the survival of untreated cells (set to 100%). **c** Similar viability assays were performed with the MDA-MB436 cell line cocultivated (or not) with adipocytes, treated (or not) with paclitaxel, 5-fluorouracil (5-FU), or mafosfamide at the indicated doses. Abbreviations: *C* cocultivated cells, *NC* non-cocultivated cells.

To address whether adipocytes play a role in promoting breast cancer resistance to DOX, a panel of estrogen receptor (ER)-positive (T47D), HER2-positive (MDA-MB453, BT-474), and triple-negative (TN) (MDA-MB436, MDA-MB231, M-Wnt, and E0771) human and murine breast cancer cell lines was cocultivated (or not) with adipocytes. Of note, the phenotype of E0771, which is generally considered an ER-positive cell line, was recently reassigned to TN, as this does not express nuclear ER, progesterone receptor, or HER2 [33]. For this, a coculture assay previously set up in our team, which reproduces the phenotypical changes observed in human tumors, was used [3, 4, 7]. Tumor cells were grown for 3 days on Transwell inserts with (C) or without (NC) adipocytes (pre-incubation period) and then treated with DOX before incubating again with or without adipocytes for 3 days (post-incubation period) (Fig. 1a). Unless indicated, the term “cocultivated cells” refers to tumor cells grown with adipocytes for the pre- and the post-incubation period. Adipocytes caused DOX resistance in all the cell lines studied, independently of the breast cancer subtypes (Fig. 1b). In addition to DOX, cocultivated tumor cells exhibit significant resistance to other drugs conventionally used in breast cancer treatment, each with different modes of anti-tumor activity (paclitaxel, 5-FU, and mafosfamide), compared with control cells grown alone (Fig. 1c). Taken together, these compelling results show that adipocytes induce DOX resistance in breast cancer cells, resistance that is associated with an MDR phenotype.

### Adipocytes promote DOX cellular efflux in breast cancer cells independently of major ABC transporters

**Fig. 2:**
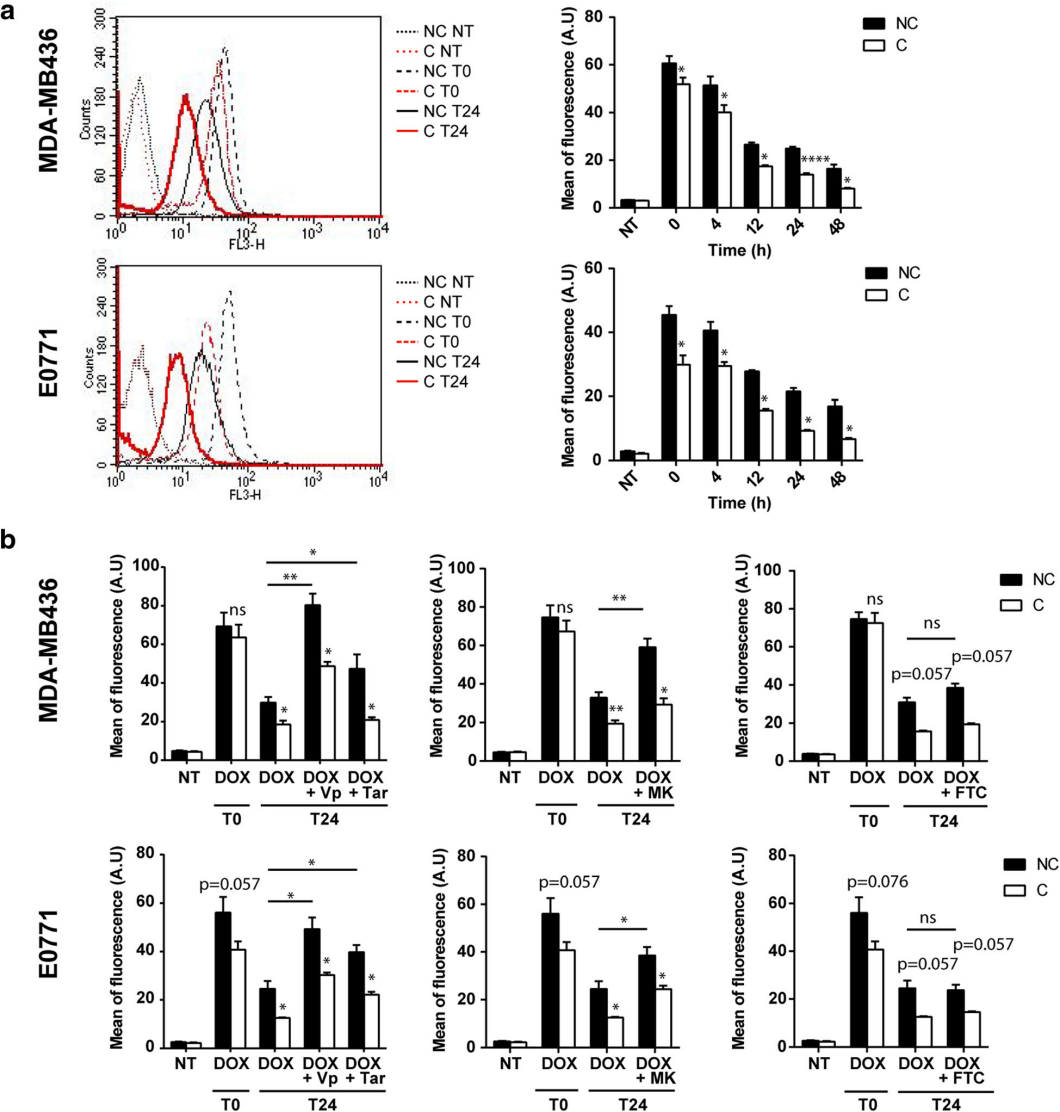
Adipocytes decrease intracellular doxorubicin accumulation in breast cancer cells independently of major ABC transporters. **a** Representative experiments showing the intracellular accumulation of doxorubicin (DOX) detected by flow cytometry for indicated times and conditions. Histograms represent the quantification of DOX intracellular fluorescence for each cell line at indicated times after drug exposure. **b** Quantification of intracellular DOX at indicated times after drug exposure, in the presence (or not) of P-gp inhibitors: verapamil (Vp) and tariquidar (Tar) (left panel), detected by flow cytometry in MDA-MB436 and E0771 cells cocultivated (C) or not (NC) with adipocytes as indicated. Similar experiments were performed by using the MRP family inhibitor, MK-571 (MK) (middle panel), and the BCRP inhibitor, Fumitremorgin-C (FTC) (right panel). Abbreviation: *a.u.* arbitrary units.

As cellular efflux mechanisms play a major role in the MDR phenotype [11], intracellular DOX accumulation was evaluated by flow cytometry at different time points after DOX treatment in cocultivated or non-cocultivated breast tumor cells (MDA-MB436 and E0771). As expected, DOX accumulation decreased from the end of the incubation with the drug (T0) in a time-dependent manner. This effect was significantly increased in cocultivated cells as compared with tumor cells grown alone (Fig. 2a). The function of the three main ABC transporters involved in DOX resistance —ABCB1 (P-gp), ABCC1 (MRP1) and ABCG2 (BCRP)— was evaluated via DOX efflux in the presence of pharmacological inhibitors in control and cocultivated cells. As expected, blocking ABCB1 (P-gp) activity with verapamil and with the highly specific inhibitor tariquidar [23] inhibits cellular efflux of DOX in MDA-MB436 cells. However, these agents were unable to reverse the differences in efflux observed between control and cocultivated cells (Fig. 2b). Similar results were obtained with the ABCC1 (MRP1) family inhibitor (MK-571) whereas the ABCG2 (BCRP) inhibitor (Fumitremorgin-C) was without significant effect on DOX cellular efflux in both control and cocultivated cells (Fig. 2b). Collectively, these results show that, despite the efficient inhibition of major ABC transporters, cocultivated cells continue to release DOX at higher rates than non-cocultivated cells and these results were confirmed in E0771 (Fig. 2b).

### Adipocytes favor DOX resistance by decreasing drug nuclear accumulation and subsequent extracellular expulsion through vesicular trafficking and secretion

**Fig. 3:**
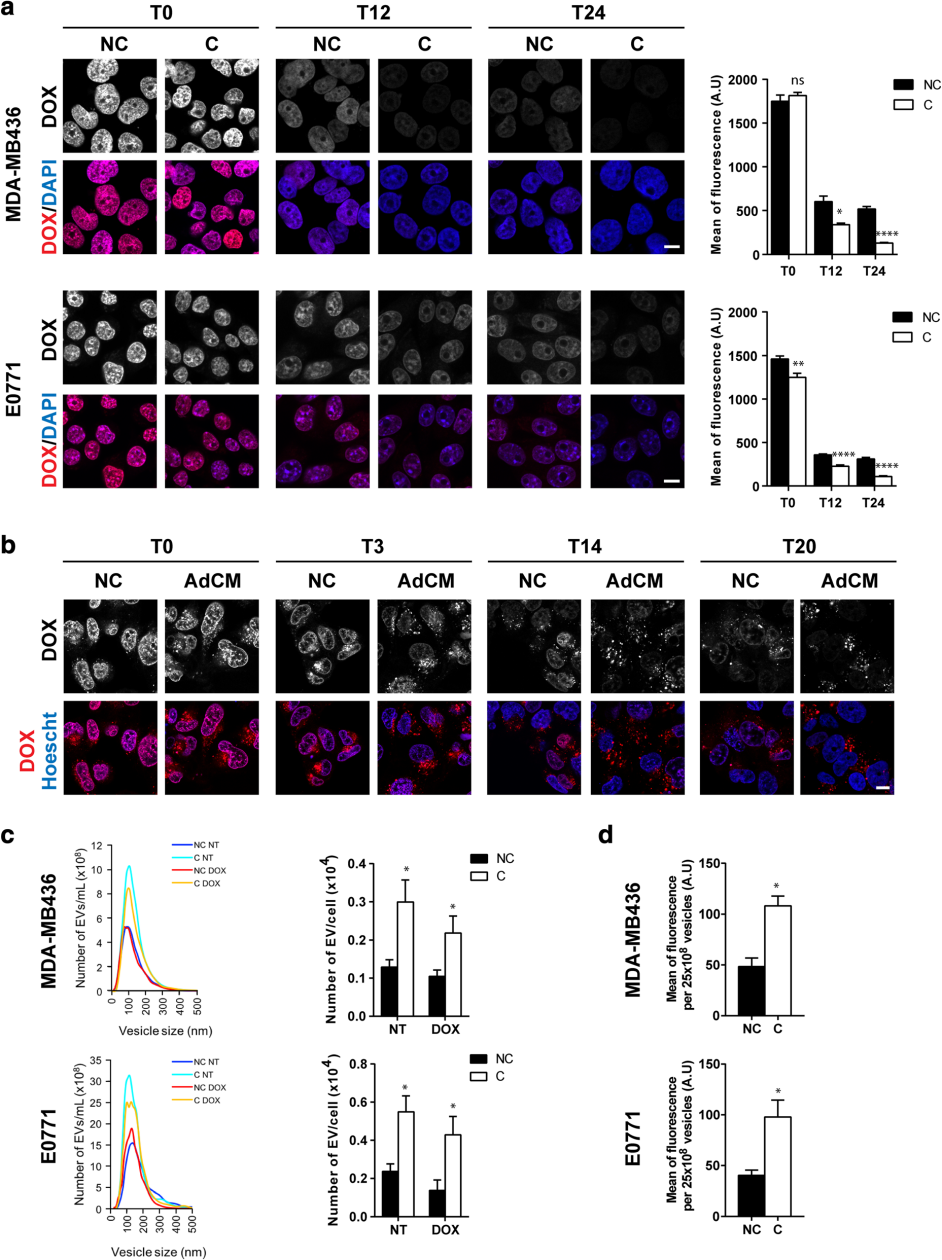
Adipocytes increase the nuclear efflux of doxorubicin (DOX) and its expulsion from tumor cells via extracellular vesicles (EVs). **a** Left panel: intracellular localization of DOX visualized by confocal microscopy in non-cocultivated (NC) and cocultivated (C) cells at indicated times after DOX exposure in MDA-MB436 and E0771 cells. Nuclei were labeled with 4′,6-diamidino-2-phenylindole (DAPI) (scale bars, 10 μm). Right panel: corresponding quantification of fluorescence intensity (DOX) in the nuclei. **b** Analysis of the cytoplasmic localization of DOX in live MDA-MB436 cells using time-lapse video microscopy in control cells (NC) and cells cultivated with adipocyte-conditioned medium (AdCM) for both the pre- and post-incubation steps at indicated times. **c** Left panel: one representative analysis using nanoparticle tracking analysis (NTA) technology of the number of EVs secreted by MDAMB436 or E0771 cells pre-incubated (C) or not (NC) with adipocytes, treated (DOX) or not (NT) and then post-incubated (C) or not (NC) with adipocyte soluble factors. Right panel: quantification of the number of EVs secreted. **d** Analysis by flow cytometry of the DOX content in EVs secreted by MDA-MB436 or E0771 cells treated as in c. Abbreviation: *a.u.* arbitrary units.

To further understand the mechanism of resistance involved, we investigated the subcellular distribution of DOX. First, nuclear accumulation of DOX was analyzed by confocal microscopy in fixed cells. Directly after drug exposure, cells showed a strong nuclear staining, and there was no visible staining in the cytoplasm in these experimental conditions. Nuclear staining declined over time (Fig. 3a), and in both cell lines (MDA-MB436 and E0771), the decrease in nuclear accumulation was enhanced by coculture with adipocytes (Fig. 3a). In fixed cells, we were unable to detect the presence of DOX within the cytoplasm. Therefore, to analyze its subcellular localization, live cells were analyzed by using time-lapse video microscopy. Rapidly after drug treatment, DOX was localized mainly in the nucleus with some early accumulation in the cytoplasm within distinct vesicle-like structures in cells incubated with AdCM (Fig. 3b). Twenty hours later, nuclear fluorescence was strongly decreased with an amplified effect in cells treated with AdCM in which almost all DOX was present in cytoplasmic vesicle-like structures (Fig. 3 b). In light of these results, we investigated whether adipocytes stimulate the sequestration of DOX within vesicles, which then could be shed into the extracellular medium. Indeed, cells are able to release such vesicles, named EVs (composed mainly of exosomes and microvesicles), which are known to be involved in mediating intercellular communication [34]. EV-mediated drug shedding has been shown to be an efflux mechanism involved in drug resistance [35, 36]. To study this process in our model, tumor cells were pre-incubated with adipocytes, treated with DOX, and then post-incubated with AdCM depleted of adipocyte-derived EVs. We previously verified that soluble factors alone reproduce the effect of total AdCM on DOX subcellular redistribution while adipocyte EVs were without effect (Additional file 4: Figure S2), thus validating the use of this fraction in our protocol. The tumor cell–derived EVs were isolated accordingly and quantified by using nanoparticle tracking analysis (NTA) technology. The number of vesicles secreted by tumor cells was increased by adipocytes by over two fold, whereas DOX treatment had no effect on EV secretion (Fig. 3c). The vesicles then were coated on aldehyde sulfate latex beads to analyze their DOX content by flow cytometry. As shown in Fig. 3d, DOX content in EVs (normalized by EV number) was significantly increased by adipocytes, implicating vesicle shedding as a drug efflux mechanism in our model. Therefore, these results show that adipocytes favor DOX efflux by increasing both the number of EVs secreted by breast tumor cells as well as the amount of DOX contained within these vesicles.

### MVP mediates adipocyte-induced DOX resistance

**Fig. 4:**
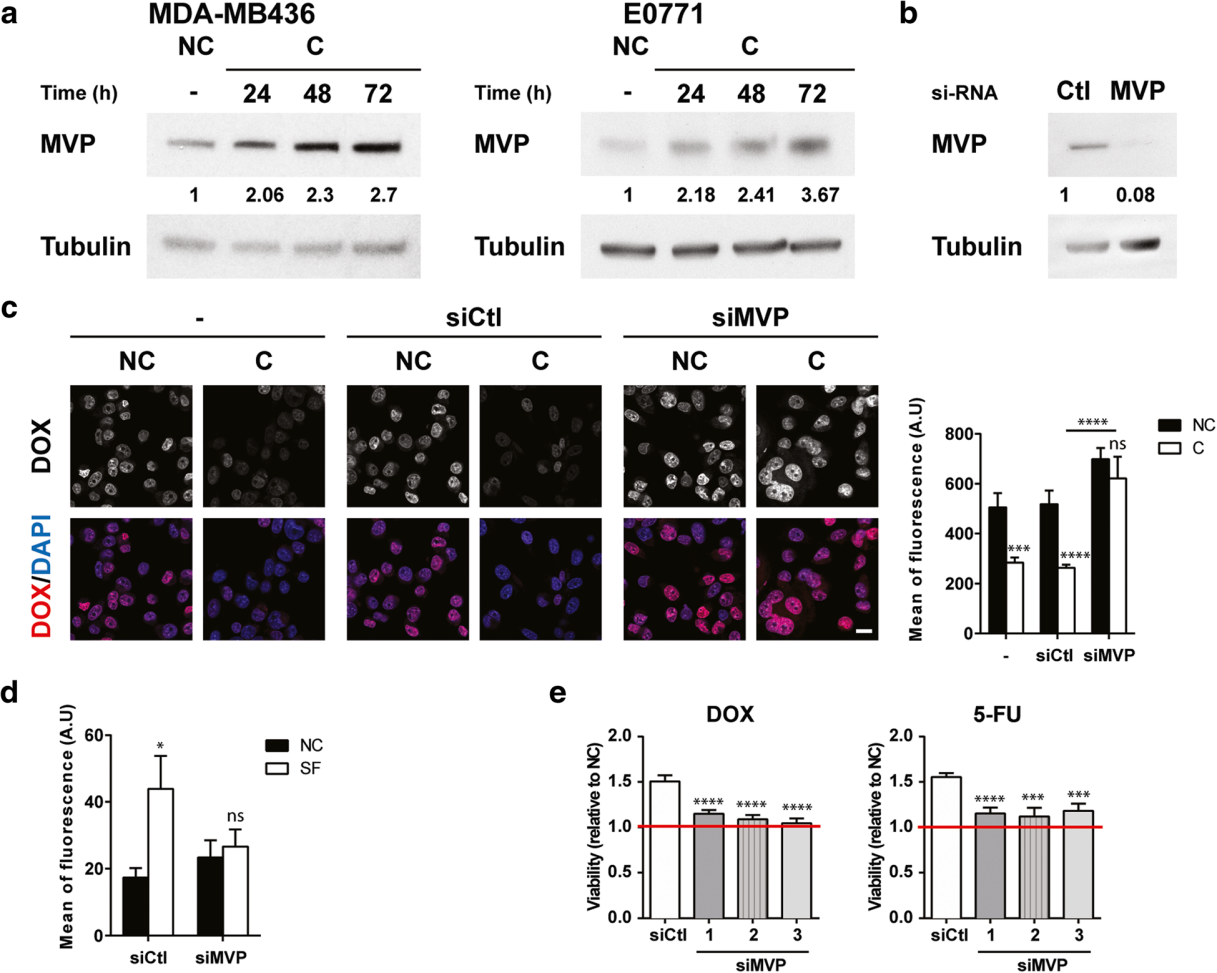
Major vault protein (MVP) is implicated in doxorubicin (DOX) efflux and mediates adipocyte-induced chemoresistance. **a** Immunoblots against MVP in MDA-MB436 (left panel) and E0771 (right panel) cells non-cocultivated (NC) or cocultivated (C) with adipocytes for the indicated times. Tubulin is shown as a control for equal protein loading. **b** MVP protein levels were analyzed after transfection of E0771 cells with Ctl and MVP small interfering RNAs (siRNA) by immunoblotting. Tubulin is shown as a control for equal protein loading. **c** Left panel: intracellular localization of DOX after transfection of E0771 cells with Ctl or MVP siRNAs visualized by confocal microscopy. Cells were post-incubated (C) or not (NC) with adipocytes for 24 h after DOX treatment. Nuclei were labeled with 4′,6-diamidino-2-phenylindole (DAPI) (scale bars, 20 μm). Right panel: corresponding quantification of fluorescence intensity (DOX) in the nuclei. **d** Analysis of DOX content in the same number of extracellular vesicles secreted by E0771 cells transfected with siCtl or siMVP and then post-incubated (or not) with adipocyte-secreted soluble factors (SF) for 20 h. **e** Analysis of cytotoxicity induced by DOX or 5-FU treatments in E0771 cells transfected by siCtl or with three independent siMVP and post-incubated with adipocyte-conditioned medium (AdCM) or not (NC) for 72 h using IncuCyte technology (expressed as the ratio of surviving cells post-incubated with AdCM over surviving NC cells). Abbreviation: *a.u.* arbitrary units.

It was previously described that vault proteins are involved in the intracellular compartmentalization of substrates, including drugs, particularly nucleocytoplasmic transport, associated with accumulation in exocytic vesicles that contribute to drug efflux [21]. The expression of MVP is increased by two fold as early as 24 h after coculture with adipocytes in E0771 and MDA-MB436 cells (Fig. 4a) and this increase in expression was maintained in DOX-treated cells (Additional file 5: Figure S3a). MVP expression is even further increased when cocultivated for a longer period (Fig. 4a). In accordance with the fact that coculture with adipocytes, rather than drug exposure, increases MVP expression in tumor cells, the levels of expression of the protein were not modified by treatment with DOX alone (Additional file 4: Figure S2b) and similar results were obtained in the presence of 5-FU and paclitaxel (Additional file 5: Figure S3b). To investigate the role of MVP in the adipocyte-induced resistant phenotype, we used an RNA silencing strategy (siRNA). The siRNA used efficiently inhibited MVP expression (Fig. 4b). To evaluate DOX nuclear accumulation in MVP-silenced cells, 24 h after transfection with either control (siCtl) or MVP (siMVP) siRNAs, cells were treated with DOX and post-incubated (or not) with adipocytes for 24 h. A strong decrease in DOX accumulation is observed in cocultivated cells compared with control cells as expected, and the effect of coculture is abolished in cells transfected with siMVP (Fig. 4c). Interestingly, the increase in DOX content in tumor EVs, induced by adipocyte-secreted SF, was abolished in cells transfected with siMVP (Fig. 4 d). We finally analyzed adipocyte-induced drug resistance in control and MVP-depleted cells, an event that represents the final end-point of the deleterious crosstalk between the two cell populations. To this end, tumor cells transfected with control or MVP siRNAs (that were not pre-incubated with adipocytes) were post-incubated after drug treatment with AdCM in order to use live-cell imaging and analysis platform that enables quantification of cell behavior over time. To strengthen our results at this crucial step of our study, two additional siRNAs (whose validation is presented in Additional file 6: Figure S4) were used. These conditions reproduced the DOX resistance induced by adipocytes as well as the MDR phenotype characterized by a cross-resistance to 5-FU (Fig. 4e). Adipocyte-mediated resistance to DOX, as well as to 5-FU, is reversed after MVP silencing using the three siRNAs (Fig. 4e). The role of MVP in DOX accumulation and adipocyte-mediated resistance to DOX was confirmed in the MDA-MB436 cell line (Additional file 7: Figure S5). These compelling results highlight the role of MVP in the resistance to DOX, as well as the changes in subcellular accumulation and efflux, induced by adipocytes.

### Chemoresistance mediated by human mammary adipocytes is increased by obesity

**Fig. 5:**
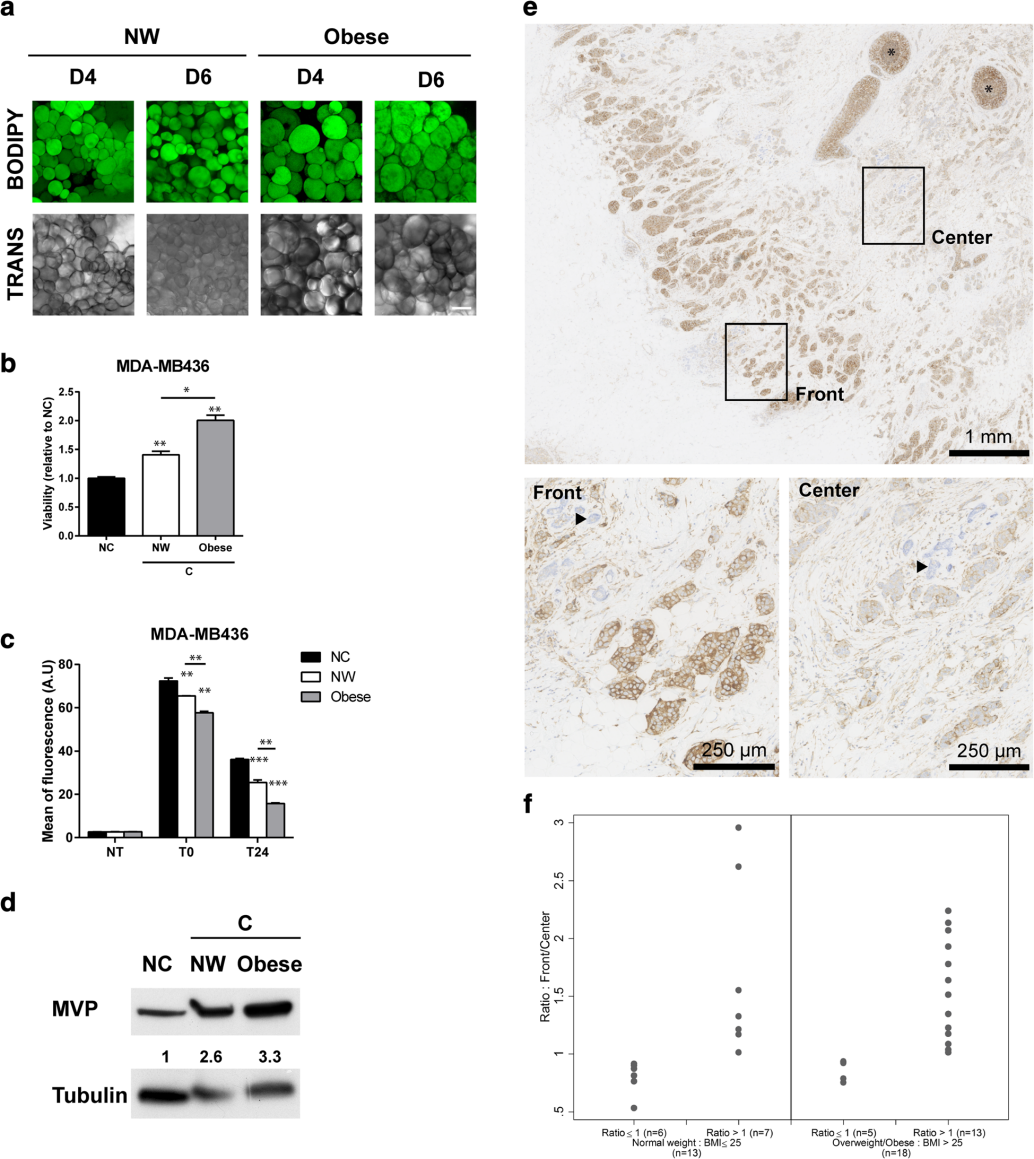
Chemoresistance is amplified by obesity and major vault protein (MVP) expression is upregulated at the invasive front of human breast tumors. **a** Neutral lipid content using bodipy staining (in green) and morphological analyses (transmission, TRANS) of mammary adipocytes embedded in a fibrinogen matrix, isolated from normal weight (NW) or obese patients (scale bars, 100 μm, pictures were taken at day 4 (D4) and day 6 (D6) of culture). Each image shows a maximum projection of about 10 pictures of a z-stack with a distance of 5 μm between each scanning position. **b** Viability assays after doxorubicin (DOX) exposure of MDA-MB436 breast cancer cell line cocultivated (C), or not (NC), with adipocytes from NW or obese women (n = 5 independent samples). Results are expressed as the ratio of surviving cells in C versus NC cells (set at 1). **c** Quantification of intracellular fluorescence of DOX in MDA-MB436 cells either non-cocultivated or cocultivated with adipocytes from NW or obese women (n = 5 independent samples) by flow cytometry at indicated times after DOX exposure. **d** Immunoblots against MVP in MDA-MB436 cells either non-cocultivated or cocultivated with adipocytes from NW or obese women. Tubulin is shown as a control for equal protein loading. Quantification of MVP is shown in arbitrary units. **e** Histologic examination of a human breast tumor (patient #15) after MVP staining (in brown); (Front) zoom of the MVP staining at the invasive front in one representative area; (Center) zoom of the MVP staining in the center of the tumor in one representative area. *In situ* carcinoma (black stars) and normal mammary glands (arrowheads) are considered positive and negative internal control, respectively. Note that the quantification did not take into account *in situ* carcinoma areas. **f** The ratio of MVP expression between the invasive front and the tumor center was calculated after quantification using Fiji software plug-ins. The results obtained in lean and overweight/obese patients are shown, and each point represents a different patient. Note that, for the clarity of the figure, two patients with ratios greater than 3 (one lean and one obese) are not represented in the figure.

As obesity is associated with poor outcome in patients with breast cancer [37], we next investigated whether the deleterious crosstalk between adipocytes and breast cancer, which promotes chemoresistance, could be amplified by obesity. To perform these experiments, we used MAT obtained from NW or obese women. It was previously demonstrated that isolated adipocytes in suspension remain viable for about 24 h but that, thereafter, both survival and function are profoundly altered [36]. To circumvent this problem, a 3D coculture system, in which isolated adipocytes are embedded in a fibrin matrix, was set up. As shown in Fig. 5a, adipocytes retain their round morphology in these culture conditions for up to 6 days. Coculture with mammary adipocytes from NW women significantly increased breast cancer cell viability compared with NC cells after DOX exposure and this effect was amplified in cells cocultivated with adipocytes from obese women (Fig. 5b). The increased resistance to DOX treatment was associated with an increase in DOX cellular efflux, which, again, was amplified in cells cocultivated with mammary adipocytes from obese women (Fig. 5c). Therefore, coculture with mammary adipocytes reproduces the DOX resistance as well as the alterations in drug efflux observed with adipocytes differentiated *in vitro* and these processes were amplified by obesity. Finally, MVP expression was increased in tumor cells cocultivated with mammary adipocytes as compared with control cells and this effect was more pronounced in obese conditions (Fig. 5d).

### MVP expression is increased at the invasive front of human tumors

In light of the results obtained with mammary adipocytes derived from lean or obese women, we next investigated whether MVP expression is regulated by contact with adipocytes and by obesity in human tumors. To address this critical issue, we investigated MVP expression in a series of 34 invasive breast tumors obtained from either lean (median BMI of 22.1) or overweight/obese patients (median BMI of 33.8), whose clinical characteristics are summarized in Additional file 2: Table S1. MVP expression was analyzed either in the tumor center or at the tumor border, where cancer cells are in close contact with adipocytes, the areas of interest being first selected on HE slides by the pathologist. A representative example of immunohistochemical staining for MVP (Fig. 5e) as well as the corresponding HE slides (Additional file 8: Figure S6) are shown. All of the tumors, except two, were positive for MVP expression. Selected stained areas of tumors were scanned and protein expression was quantified by using Fiji software plug-ins to obtain more accurate measures than the evaluation relying on manual scoring. As shown in Fig. 5e, we observed a positive regulation of MVP expression at the tumor border, in regions that are in close contact with adipocytes, as compared with the tumor center. A gradient of MVP expression (defined by a ratio of expression at the tumor border over the expression at the tumor center greater than 1) was present in 22 out of 34 samples (66.7% of the patients) and represented 57.1% (median ratio of 1.44) and 73.7% (median ratio of 1.58) of the samples in lean and overweight/obese women respectively (Fig. 5f and Additional file 2: Table S1). Taken together, these results highlight that MVP expression is upregulated by contact with adipocytes in human tumors with a slight, but not significant, enhancement of the effect in tumors obtained from overweight/obese patients in this pilot cohort.

## Discussion

**Fig. 6:**
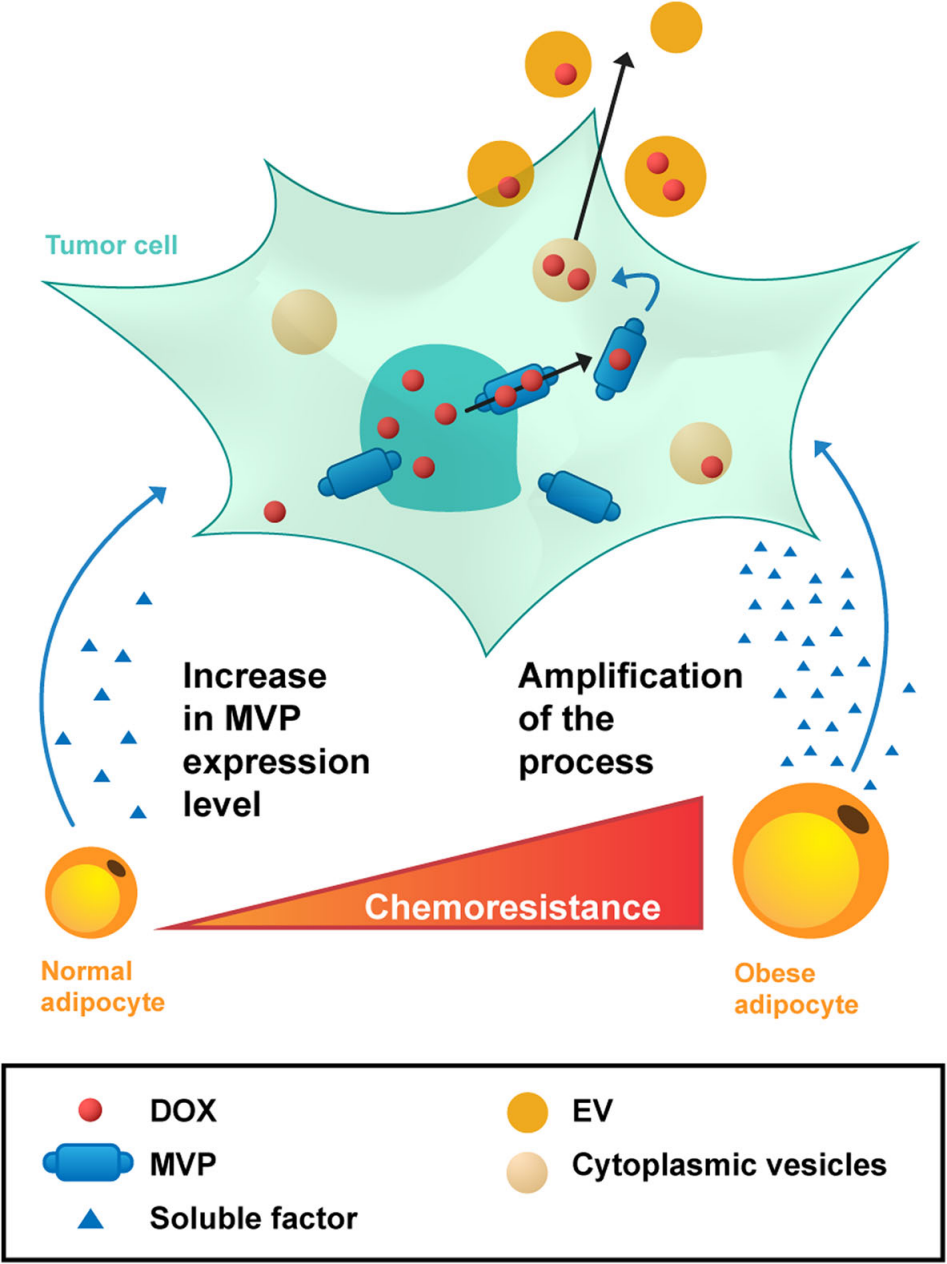
Schematic representation of the role of tumor-surrounding adipocytes in breast cancer resistance to doxorubicin (DOX). DOX enters tumor cells by passive diffusion and accumulates in the nucleus. Upon stimulation with adipocyte soluble factors, the level of major vault protein (MVP) increases, promoting DOX nuclear efflux. This efflux is followed by sequestration of DOX in cytoplasmic vesicles, and extracellular efflux of the drug is mediated through secretion of these vesicles (extracellular vesicles, EV). This adipocyte-mediated mechanism contributes to the occurrence of a resistant phenotype that is amplified by obesity

Our work reveals a new role for tumor-surrounding adipocytes, which confer an MDR phenotype to breast cancer cells, and the associated mechanistic pathway that is summarized in Fig. 6. In the presence of adipocytes, DOX, which accumulates in the nucleus where it binds to DNA, is exported from the nucleus through an MVP-dependent process to cytoplasmic vesicles. Tumor cells then are able to secrete these DOX-containing vesicles into the extracellular medium. In MVP-depleted cells, this process is reversed. Soluble factors secreted by adipocytes regulate this process. Interestingly, this mechanism is amplified by obesity, which could explain, at least in part, the increased chemoresistance observed in this subset of patients. Our findings were confirmed in invasive human breast tumors, in which the tumor cells that are in close proximity to adipocytes express higher levels of MVP compared with those found in the tumor center.

Our results demonstrate that adipocytes modify DOX subcellular distribution in tumor cells with decreased nuclear accumulation, subsequent accumulation in cytoplasmic vesicles, and efflux into the extracellular medium through EV secretion. This process is mediated by MVP, the main component of ubiquitous, large cellular ribonucleoparticles termed vaults, which makes up more than 70% of the vault mass (for review, see [38]). MVP is capable of generating the minimal vault structure and, because it is found only in vaults, knockdown of MVP in cells is regarded as equivalent to vault knockdown (for review, see [38]). Vaults have been implicated in chemoresistance but the association remains largely correlative [17, 18] and their exact mechanism of action remains unclear. Using a gene silencing strategy, we have shown that MVP decreases DOX nuclear accumulation (Fig. 4c), thereby preventing the interaction with its main target, DNA. Despite predominant expression of vaults in the cytoplasm, a subgroup of vaults has repeatedly been reported to be localized in the nuclear envelope of human cells where they could play a shuttle function between the nucleus and the cytoplasm (for review, see [38, 39]). In addition to our study, only a few studies report that MVP is directly implicated in regulating DOX nucleocytoplasmic transport. Indeed, in a human colon cancer cell line, sodium butyrate treatment induced an MDR phenotype that was based on the nuclear exclusion of DOX, and transfection with an MVP-targeting ribozyme restored chemosensitivity and nuclear drug accumulation [40]. Comparable effects were seen when isolated nuclei were incubated with an anti-MVP antibody [40]. More recently, it was demonstrated that depletion of MVP increases DOX sensitivity and nuclear accumulation in a bladder cancer cell line [41]. Despite these data, questions remain concerning the fate of DOX once sequestered in the cytoplasm. Accumulation of DOX in a vesicular compartment within the cytoplasm of tumor cells, identified mostly as lysosomes, has been largely documented [42, 43] and the role of MVP in this process has been demonstrated [41]. Cellular drug efflux into the extracellular medium could occur either through direct secretion of exocytic vesicles or in cooperation with ABC transporters or both [21], although direct experimental evidence is lacking. By purifying EVs and analyzing their DOX content, we were able to demonstrate that downregulation of MVP is a necessary and sufficient event to inhibit the process of DOX efflux within vesicles (Fig. 4d). The signal emanating from adipocytes that regulates DOX subcellular distribution is a soluble factor (Additional file 4: Figure S2 and Fig. 4d). We recently demonstrated that MVP is present at high levels in the exosomes secreted by adipocytes [44], but purified vesicles were not able to reproduce the altered distribution of DOX (Additional file 4: Figure S2). The nature of the soluble factor(s) involved in the regulation of MVP expression requires further investigation, but the great diversity of the adipocyte secretome, as well as the lack of available data regarding factors regulating MVP expression, renders this task particularly difficult. It has been described that interferon-gamma (IFNγ) is able to upregulate MVP promoter activity and its mRNA level [45]. However, in AT, IFNγ is secreted by recruited inflammatory cells, and not by adipocytes, especially in obesity [46]. Finally, we demonstrate that, in addition to DOX, coculture with adipocytes contributes to resistance to 5-FU, paclitaxel, and mafosfamide, other drugs used in breast cancer treatment. All of these drugs have been reported to be potential substrates of MVP [47–49], and the reversal of drug resistance to DOX and 5-FU agents upon MVP expression highlights the involvement of this protein in generating the MDR phenotype observed (Fig. 4e).

We further demonstrated that obesity amplifies the MVP-associated phenotype (Fig. 5). Obesity is associated with infiltration of AT by inflammatory macrophages, hypertrophy of adipocytes, and changes in their secretory profiles [50]. We have recently demonstrated that these changes occur in MAT, even in overweight patients [51]. In our *in vitro* coculture system using human mammary adipocytes, MVP expression was increased in the presence of adipocytes isolated from obese compared with lean patients. This was associated with a clear increase in DOX resistance and amplified cellular efflux (Fig. 5). In the series of patients where MVP expression was investigated by immunohistochemistry, we observed a slight but not significant enhancement of MVP expression at the tumor border (compared with the tumor center) in human breast cancers from overweight/obese as compared with lean patients. This absence of significant differences could be due to the small size of our cohort and also to the fact that 75% of the patients were overweight to moderately obese (BMI between 25 and 35 kg/m^2^). In addition, as inflammation of MAT is also observed in a subset of patients with normal BMIs [52], an extensive characterization of MAT status (in addition to the measure of BMI) in relation to MVP expression is crucial for further studies.

One very important aspect of our study is its clinical relevance. First, it has to be noted that expression of MVP is observed in MDR cell lines with low or intermediate levels of resistance, like in our study (Fig. 1b–c), and subsequent upregulation of ABCB1 occurred only in cell lines that exhibited high levels of resistance [53]. These findings suggest that MVP expression could be used as a marker for low levels of drug resistance, which is clinically relevant. Another important finding is the fact that a gradient of MVP expression is observed between the invasive front and the tumor center of human breast tumors (Fig. 5e), highlighting that the depicted mechanism observed *in vitro* also exists *in vivo*. In our previous studies, aimed at deciphering the crosstalk between adipocytes and breast cancer cells, we have consistently shown that the observed changes occur only at the invasive front. Therefore, a close proximity between the two populations is needed for these changes to occur in human cancers [3]. Expression of MVP in these tumor cells is particularly important because the cells present at the tumor border and within the stroma are more likely to disseminate [54] to give rise to chemoresistant metastasis. These data could also explain the inconclusive results regarding the predictive value of MVP expression in the chemotherapy response of human breast cancers [55, 56], as the expression level was analyzed in the core of the tumors.

## Conclusion

To the best of our knowledge, this study is the first to report a direct role of adipocytes in regulating drug response in breast cancer cells; the MDR phenotype observed highlights its potential importance at clinical levels. Implication of MVP in the clinical response to chemotherapy now needs to be confirmed using a dedicated collection that includes the tumor border in the studied samples. If these results were confirmed, they would clearly reinforce the interest for the development of MVP inhibitors that for the moment are lacking.

## Additional files

Additional file 1: Supplementary materials and methods. (PDF 142 kb)

Additional file 2: **Table S1.** Clinical characteristics of the patients with breast cancer and histopathological characteristics of the breast tumors. (PDF 138 kb)

Additional file 3: **Figure S1.** Detection of lung resistance protein/major vault protein (LRP/MVP) by immunohistochemistry in formalin-fixed paraffin-embedded normal human tissue. (PDF 698 kb)

Additional file 4: **Figure S2.** Adipocyte soluble factors reproduce the effect of adipocyte-conditioned medium (AdCM) on subcellular redistribution of doxorubicin (DOX) in tumor cells. (PDF 237 kb)

Additional file 5: **Figure S3.** Major vault protein (MVP) expression is not regulated by exposure to drugs. (PDF 124 kb)

Additional file 6: **Figure S4.** Validation of the two additional small interfering RNAs (siRNAs) targeting major vault protein (MVP). (PDF 94 kb)

Additional file 7: **Figure S5.** Major vault protein (MVP) is implicated in doxorubicin (DOX) efflux and mediates adipocyte-induced chemoresistance in the human MDA-MB436 cell line. (PDF 282 kb)

Additional file 8: **Figure S6.** Hematoxylin/eosin staining of the tumor used to represent major vault protein (MVP) expression (Fig. 5e). (PDF 670 kb)

## Abbreviations

3D: Three-dimensional
5-FU: 5-fluorouracile
ABC: ATP-binding protein
ABCB1: Pgp, P-glycoprotein
ABCC1: MRP1, multidrug resistance-associated protein 1
ABCG2: BCRP, breast cancer resistance protein
AdCM: Adipocyte-conditioned medium
AT: Adipose tissue
BCRP: Breast cancer resistance protein
BMI: Body mass index
DAPI: 4′,6-diamidino-2-phenylindole
DMEM: Dulbecco’s modified Eagle’s medium
DOX: Doxorubicin
ER: Estrogen receptor
EV: Extracellular vesicle
FCS: Fetal calf serum
HE: Hematoxylin and eosin
IFNγ: Interferon gamma
LRP: Lung resistance protein
mAb: Monoclonal antibody
MAT: Mammary adipose tissue
MDR: Multidrug resistance
MVP: Major vault protein
NW: Normal weight
PBS: Phosphate-buffered saline
PFA: Paraformaldehyde
SF: Soluble factor
siMVP: Small interfering MVP
siRNA: Small interfering RNA
TN: Triple-negative
