# Supplementary material for: Adipocytes promote breast cancer resistance to chemotherapy, a process amplified by obesity: role of the major vault protein (MVP)
Source: Breast Cancer Res. 2019 Jan 17;21:7. doi: 10.1186/s13058-018-1088-6 (PMC6337862; doi:10.1186/s13058-018-1088-6)
Supplement: Supplementary file 1 — Supplementary materials and methods. (PDF 142 kb) [file 13058_2018_1088_MOESM1_ESM.pdf]

Supplementary materials and methods

Adipocyte-derived extracellular vesicles (EVs) and soluble factors (SF) preparation The conditioned medium of adipocytes was prepared as follows: 3T3-F442A adipocytes were cultivated for 48 hours with complete DMEM medium without phenol red (to avoid fluorescence contamination during DOX analysis in tumor cells) previously depleted of contaminating bovine vesicles by overnight centrifugation at 100,000g. This medium was then collected and centrifuged at 3,000 g for 30 minutes to eliminate cell debris contamination (AdCM). To obtain the different fractions of AdCM, this one was first centrifuged for 60 minutes at 10,000 g. The obtained pellet contains microvesicles (MV) and the supernatant contains exosomes and SF. The supernatant was then ultracentrifuged for 75 minutes at 100,000g to isolate exosomes (pellet) and the resulting supernatant was again ultracentrifuged overnight at 100,000 g to obtain SF fraction. Exosomes and MV were suspended together in DMEM medium, previously depleted of bovine vesicles, to obtain the total EVs fraction. Tumor cells were incubated with AdCM, SF fraction or EVs purified from an equivalent quantity of this conditioned medium.

Clinical study design Thirty four women between 35 and 74 years of age, diagnosed with histologically verified invasive breast cancers, were selected at random between 1st October 2014 and 31st March 2016 for this pilot clinical study at the "Institut Universitaire du Cancer de Toulouse". All participants signed an informed consent form. All breast tumors were histologically examined and classified according to the invasive histological type (ductal, lobular), histological grade (I-III), and tumor diameter (both macro- and microscopically, in mm). Axillary lymph nodes were analyzed to assess for metastasis, using sentinel lymph node (SN) technique or axillary lymphadenectomy. Tumors were analyzed using immunohistochemistry against selected markers: estrogen receptors (ER), progesterone receptor (PgR - clone 636), human epidermal growth factor receptor 2 (HER2), and tumor cell proliferation index (Ki67), markers that are routinely used. Hormone receptor expression was given as the average percentage of positive cells in the tumor. ER positive status was defined as ≥10% ER-expressing tumor cells, and PgR positive status as ≥10% PgR-expressing tumor cells. The assessment of HER2 status was performed according to the ASCO/CAP guidelines. The percentage of Ki67 positive tumor cells was determined according to national and international guidelines. The following antibodies were used: ER (clone EP1, Dako, Glostrup, Denmark), PgR (clone 636, Dako, Glostrup, Denmark), HER2/neu (clone 4B5, Roche Diagnostics, Mannheim, Germany), and Ki67 (clone MIB1, Roche Diagnostics, Mannheim, Germany). HER2 *in situ* hybridization was performed using HER2 Dual ISH kit (Roche Diagnostics, Mannheim, Germany). ER and PgR immunostaining were performed on AutoStainer Link 48 (Dako, Glostrup, Denmark). HER2/neu and Ki67 immunostaining and HER2 *in situ* hybridization were performed on a Benchmark Ultra (Ventana, Tucson, AZ).

RNA interference

Transient transfection of MDA-MB436 cells with human small interfering RNAs (hsiRNA) was performed with Lipofectamine® RNAiMAX Reagent according to the manufacturer's instructions. Transfection was performed in 6-well plates containing 50% confluent cells in appropriate medium to which the transfection mix was added to a final siRNA concentration of 100 nM. Five hours after transfection, medium was replaced with fresh DMEM containing 10% FCS. A second transfection was performed 24h after the first one. The 2 siRNAs directed against human MVP used were from Eurofins Genomics (Ebersberg, Germany). Their target sequences are as follows: hsiMVP1 (also named siMVP), 5'-AUCAUUCGCACUGCUGU-3'; hsiMVP2, 5'-UAGGAGUCACCAUGGCAAC-3'). Control siRNA containing an untargeted sequence was provided by Qiagen (SI1022076). To analyze MVP protein level by western blot, cells were incubated with normal medium during 48h then trypsinized.

DOX intranuclear accumulation analysis in MDA-MB436 cell line

For measure of DOX intranuclear accumulation, tumor cells transfected with 100 nM siRNA control (siCtl) or directed against MVP (siMVP) grown on glass coverslips, incubated with normal medium (NC) or adipocytes conditioned medium (AdCM) were treated with DOX then analysed following the protocol described in materials and methods.

Viability experiments

MDA-MB436 cells transfected with siRNAs control (Ctl) or directed against MVP (MVP1, MVP2) were seeded into 96-well plates (3.5×10^3/well) in complete DMEM medium and incubated overnight before replacing with fresh medium containing DOX for 4 hours. After treatment, fresh DMEM medium or AdCM were added and plates were transferred to the IncuCyte for confluence analysis. Data shown are from 3 replicates (2 images per time-point).

Statistical analysis Comparisons between groups were performed using Mann–Whitney U test. The Benjamini-Hochberg procedure was applied for multiple comparisons. All reported p-values were two-sided. For all statistical tests, differences were considered significant at the 5% level. Statistical analysis was performed using R 3.2.2 software. Bar and errors flags represent means ± SEM of at least 3 independent experiments.
