# Supplementary material for: Adipocytes promote breast cancer resistance to chemotherapy, a process amplified by obesity: role of the major vault protein (MVP)
Source: Breast Cancer Res. 2019 Jan 17;21:7. doi: 10.1186/s13058-018-1088-6 (PMC6337862; doi:10.1186/s13058-018-1088-6)
Supplement: Supplementary file 2 — Table S1. Clinical characteristics of the patients with breast cancer and histopathological characteristics of the breast tumors. (PDF 138 kb) [file 13058_2018_1088_MOESM2_ESM.pdf]

Table S1. Clinical characteristics of the breast cancer patients and histopathological characteristics of the breast tumors

Characteristics	Total	BMI		
	(n=34)[a]	<25 kg/m^2 (n=15)[a]	$\geq$25 kg/m^2 (n=19)[a]	***P*-value**
Age at diagnosis, years	58.5 (35-74)	52 (35-74)	60 (37-72)	***0.041***
BMI, kg/m^2	26.0 (20.0-46.1)	22.1 (20.0-24.8)	33.8 (25.4-46.1)	***<0.001***
**Comorbidities**				
Diabetes, %				*0.053*
No	29 (85.3)	15 (100.0)	14 (73.7)	
Yes	5 (14.7)	0 (0.0)	5 (26.3)	
Hypertension, %				***0.005***
No	26 (76.5)	15 (100.0)	11 (57.9)	
Yes	8 (23.5)	0 (0.0)	8 (42.1)	
Hypercholesterolemia, %				*0.113*
No	30 (88.2)	15 (100.0)	15 (78.9)	
Yes	4 (11.8)	0 (0.0)	4 (21.1)	
**Surgical treatment**				
Conservative surgery, %	19 (55.9)	7 (46.7)	12 (63.2)	*0.336*
Mastectomy, %	15 (44.1)	8 (53.3)	7 (36.8)	
Sentinel lymph node	20 (58.8)	9 (60.0)	11 (57.9)	*0.717*
Axillary lymphadenectomy	13 (38.2)	5 (33.3)	8 (42.1)	
**Breast tumor and lymph node characteristics**				
Invasive ductal carcinoma, %	32 (94.1)	15 (100.0)	17 (89.5)	*0.492*
Invasive lobular carcinoma, %	2 (5.9)	0 (0.0)	2 (10.5)	
Tumor diameter, mm	17.0 (7.0-65.0)	17.0 (10.0-35.0)	18.0 (7.0-65.0)	*0.742*
Grade, %				
1	3 (8.8)	1 (6.7)	2 (10.5)	*0.578*
2	21 (61.8)	8 (53.3)	13 (68.4)	
3	10 (29.4)	6 (40.0)	4 (21.1)	
Ki67 hotspot, (n=23), %	20 (5-30)	20 (5-25)	17.5 (5-30)	*0.682*
Hormone receptor positive, %	30 (88.2)	13 (86.7)	17 (89.5)	*1.000*
HER2 positive, %	6 (17.6)	3 (20.0)	3 (15.8)	*1.000*
Lymph node				
N0	17 (50.0)	12 (80.0)	5 (26.3)	***0.002***
N+	17 (50.0)	3 (20.0)	14 (73.7)	
**MVP analysis**				
Ratio Front/Center (n=33)				*0.459*
>1, %	22 (66.7)	8 (57.1)	14 (73.7)	
<1, %	11 (33.3)	6 (42.9)	5 (26.3)	
Median ratio > 1 (n=22)	1.53 (1.01-9.27)	1.44 (1.01-9.27)	1.58 (1.01-5.73)	*0.838*

The data are presented for the overall cohort and stratified by BMI; presented as median (range) or n (%).

[a] Number of patients may vary due to missing information

Abbreviations: BMI, body mass index (kg/m^2); Hormone receptor (estrogen receptor positive when ≥ 10% and progesterone receptor positive when ≥ 10%); HER2, human epidermal growth factor receptor 2; MVP: Major Vault Protein.
