# Supplementary material for: Adipocytes promote breast cancer resistance to chemotherapy, a process amplified by obesity: role of the major vault protein (MVP)
Source: Breast Cancer Res. 2019 Jan 17;21:7. doi: 10.1186/s13058-018-1088-6 (PMC6337862; doi:10.1186/s13058-018-1088-6)
Supplement: Supplementary file 3 — Figure S1. Detection of lung resistance protein/major vault protein (LRP/MVP) by immunohistochemistry in formalin-fixed paraffin-embedded normal human tissue. (PDF 698 kb) [file 13058_2018_1088_MOESM3_ESM.pdf]

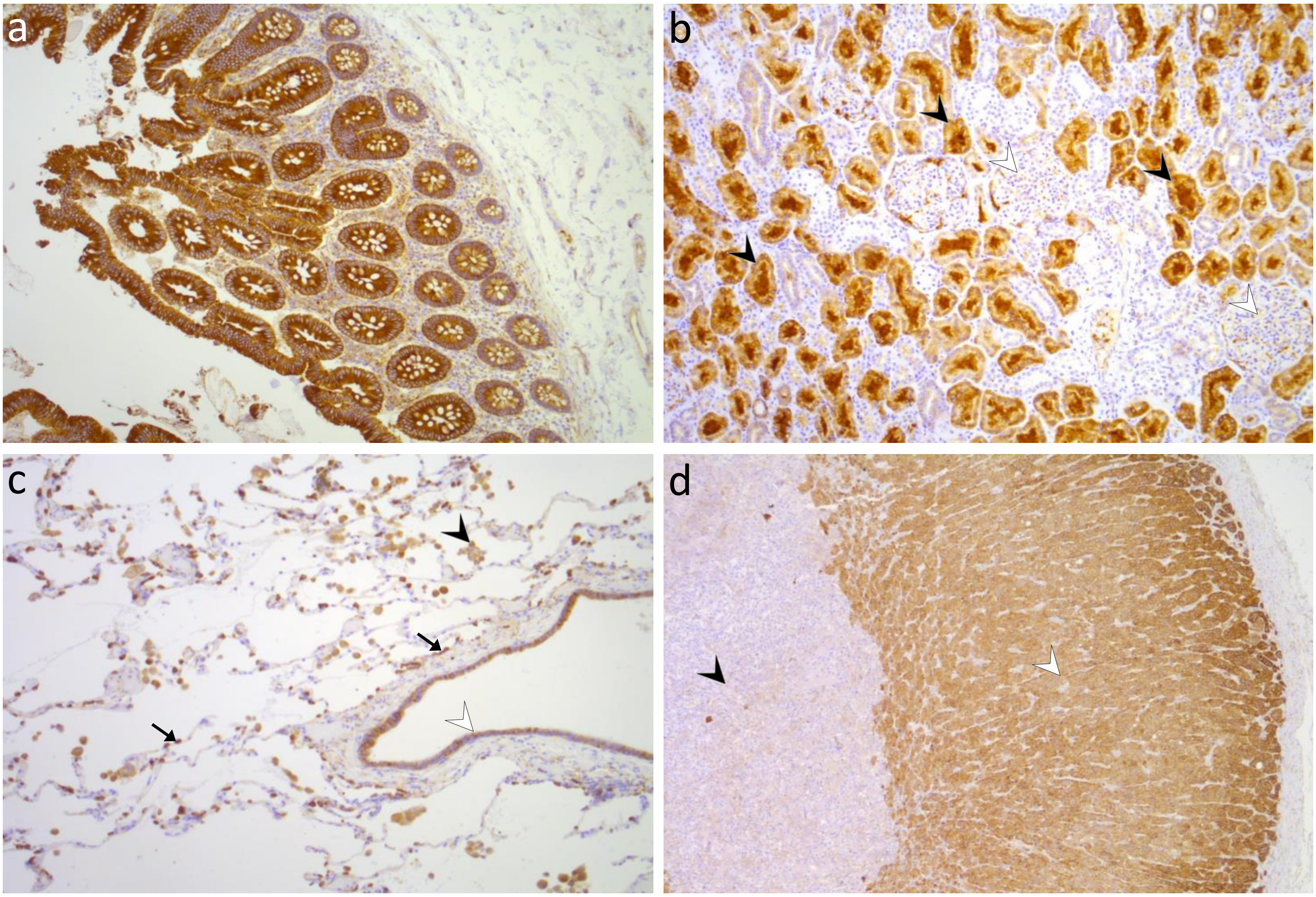

Figure S1. Detection of LRP/MVP by immunohistochemistry in formalin-fixed paraffin-embedded normal human tissue.

a Gastrointestinal tract. Cytoplasmic staining of colonic epithelial cells with gradient of staining from surface epithelium toward lowermost parts of the crypts (magnification x 100). **b** Kidney. Intense cytoplasmic staining of proximal tubules (black arrows heads) and lack of staining of glomeruli (white arrows heads) (magnification x 100). **c** Lung. Cytoplasmic staining of bronchial cells (white arrow head), alveolar macrophages (black arrow head) and pneumocytes (black arrows) (magnification x 100). **d** Adrenal gland. Cytoplasmic staining of the cortex (white arrow head) and lack of staining of medulla (black arrow head) (magnification x 50).
