# Supplementary material for: Adipocytes promote breast cancer resistance to chemotherapy, a process amplified by obesity: role of the major vault protein (MVP)
Source: Breast Cancer Res. 2019 Jan 17;21:7. doi: 10.1186/s13058-018-1088-6 (PMC6337862; doi:10.1186/s13058-018-1088-6)
Supplement: Supplementary file 4 — Figure S2. Adipocyte soluble factors reproduce the effect of adipocyte-conditioned medium (AdCM) on subcellular redistribution of doxorubicin (DOX) in tumor cells. (PDF 237 kb) [file 13058_2018_1088_MOESM4_ESM.pdf]

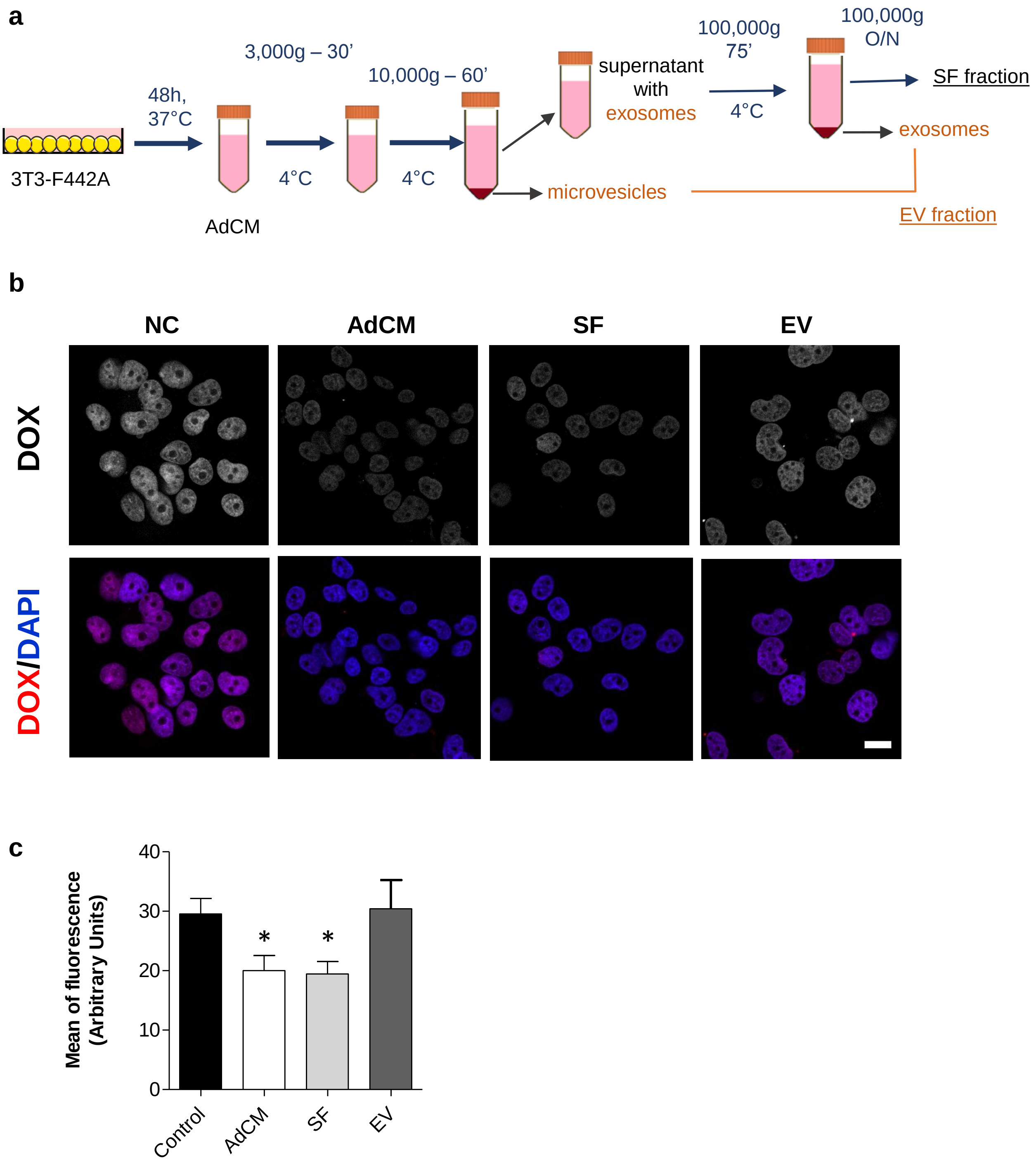

Figure S2. Adipocyte soluble factors reproduce the effect of AdCM on subcellular redistribution of DOX in tumor cells. **a** Experimental protocol used to obtain soluble factors (SF) and extracellular vesicles (EV) fractions: the conditioned medium of 3T3-F442A adipocytes (AdCM) was prepared by incubating cells in ultracentrifuged DMEM medium supplemented with 10% SVF for 48h. The obtained medium was first centrifuged at 3,000 g for 30 min to remove debris and apoptotic bodies. To separate vesicles (indicated in orange in the figure), AdCM was then centrifuged for 60 minutes at 10,000g (to pellet microvesicles) and 75 minutes at 100,000g (to pellet exosomes). After an additional centrifugation (100,000g overnight (O/N)), the vesicle-depleted supernatant therefore contains only SF. Microvesicles and exosomes successively pelleted by centrifugation were resuspended in ultracentrifugated DMEM (EV fraction). SF and EV were kept at −80 °C until use. **b** Intracellular localization of DOX visualized by confocal microscopy in noncocultivated (NC) MDA-MB436 cells or treated with AdCM or the different indicated fractions for 24h after DOX exposure (scale bars, 20 μm). **c** Corresponding quantification of fluorescence intensity (DOX) in the nuclei.
