# Supplementary material for: Adipocytes promote breast cancer resistance to chemotherapy, a process amplified by obesity: role of the major vault protein (MVP)
Source: Breast Cancer Res. 2019 Jan 17;21:7. doi: 10.1186/s13058-018-1088-6 (PMC6337862; doi:10.1186/s13058-018-1088-6)
Supplement: Supplementary file 5 — Figure S3. Major vault protein (MVP) expression is not regulated by exposure to drugs. (PDF 124 kb) [file 13058_2018_1088_MOESM5_ESM.pdf]

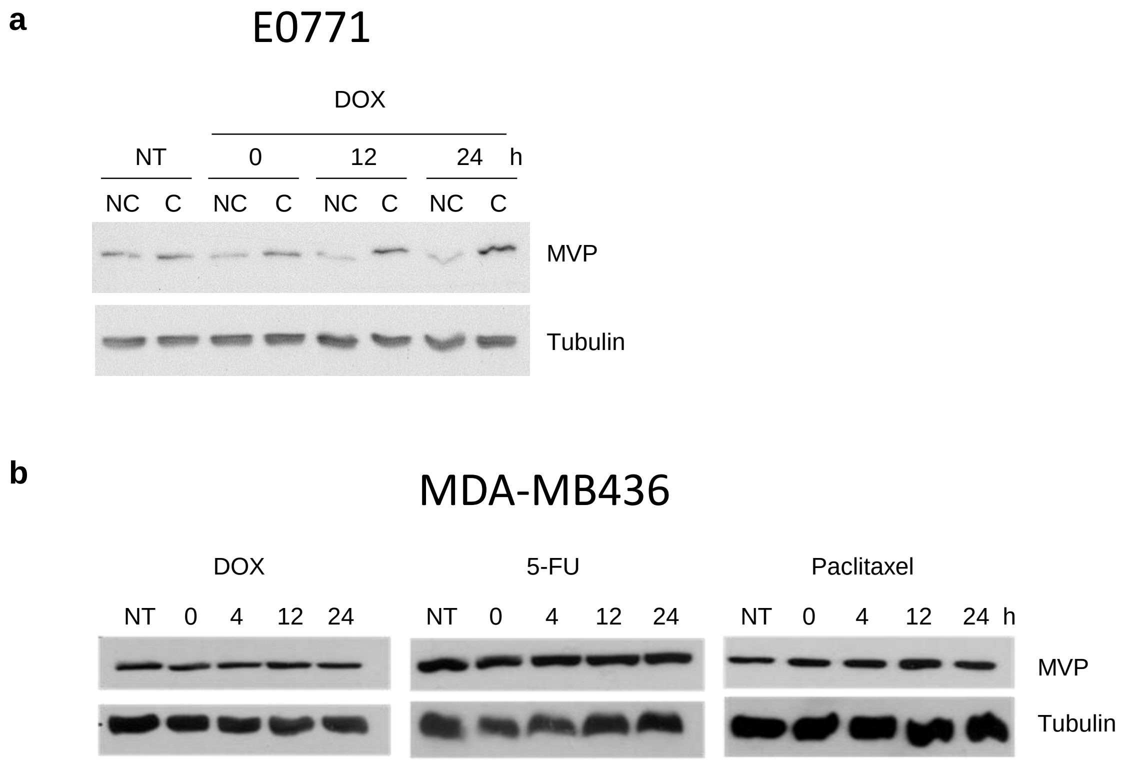

Figure S3. MVP expression is not regulated by exposure to drugs. **a** E0771 cells were cocultivated (C) or not (NC) with adipocytes and treated or not (NT) with DOX. Proteins were extracted just at the end of drug treatment (0h) or at indicated times after drug exposure. Immunoblots against MVP were performed. Tubulin is shown as a control for equal protein loading. **b** MDA-MB436 were exposed or not (NT) to the indicated drugs and proteins were extracted at indicated times after drug exposure. Tubulin is shown as a control for equal protein loading. The following drug concentrations were used : DOX (2 µg/mL), 5-FU (4 µg/mL), Paclitaxel (200 µM).
