# Supplementary material for: Adipocytes promote breast cancer resistance to chemotherapy, a process amplified by obesity: role of the major vault protein (MVP)
Source: Breast Cancer Res. 2019 Jan 17;21:7. doi: 10.1186/s13058-018-1088-6 (PMC6337862; doi:10.1186/s13058-018-1088-6)
Supplement: Supplementary file 6 — Figure S4. Validation of the two additional small interfering RNAs (siRNAs) targeting major vault protein (MVP). (PDF 94 kb) [file 13058_2018_1088_MOESM6_ESM.pdf]

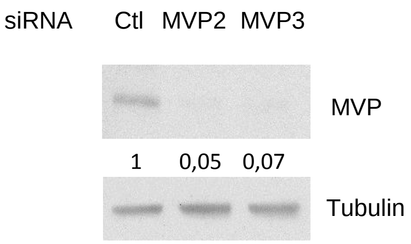

Figure S4. Validation of the two additional siRNAs targeting MVP. E0771 cells were transfected with control (Ctl) siRNA or with two siRNAs directed against MVP (MVP2, MVP3) (see Material and methods for their target sequences). Forty eight hours after transfection, proteins were extracted and the expression of MVP was assessed by Western blot. Tubulin is shown as a control for equal protein loading.
