# Supplementary material for: Adipocytes promote breast cancer resistance to chemotherapy, a process amplified by obesity: role of the major vault protein (MVP)
Source: Breast Cancer Res. 2019 Jan 17;21:7. doi: 10.1186/s13058-018-1088-6 (PMC6337862; doi:10.1186/s13058-018-1088-6)
Supplement: Supplementary file 7 — Figure S5. Major vault protein (MVP) is implicated in doxorubicin (DOX) efflux and mediates adipocyte-induced chemoresistance in the human MDA-MB436 cell line. (PDF 282 kb) [file 13058_2018_1088_MOESM7_ESM.pdf]

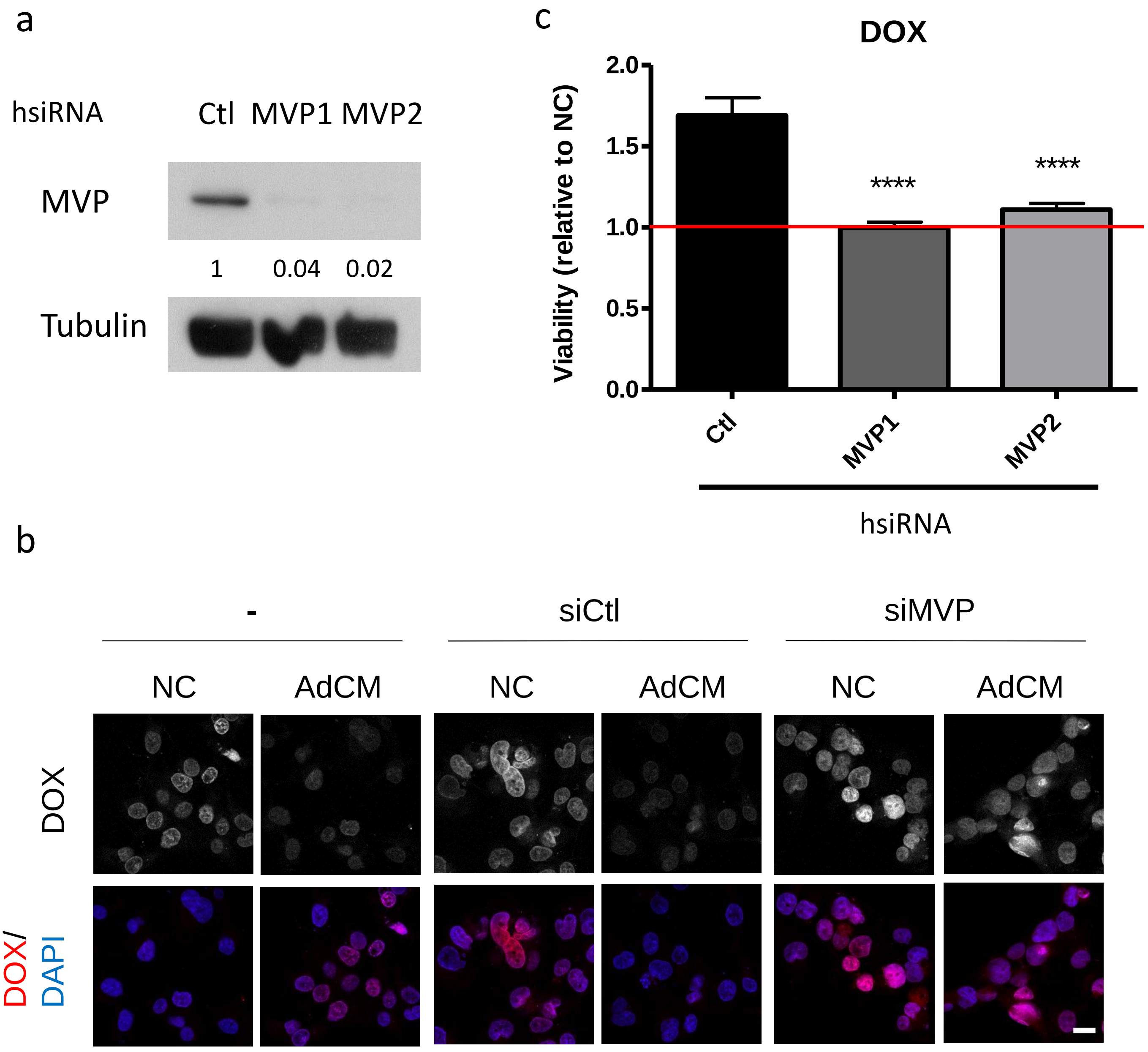

Figure S5. MVP is implicated in DOX efflux and mediates adipocyte-induced chemoresistance in the human MDA-MB436 cell line.

a Immunoblots against MVP in MDA-MB436 cells transfected with human siRNAs (hsiRNA). Protein levels were analyzed after transfection of cells with control (Ctl) or with two siRNAs directed against MVP (MVP1, MVP2). Tubulin is shown as a control for equal protein loading. **b** Intracellular localization of DOX after transfection (or not, -) of cells with control (siCtl) or MVP2 hsiRNAs (siMVP) visualized by confocal microscopy. Cells were post-incubated with adipocytes conditioned medium (AdCM) or not (NC) for 24h after DOX treatment. Nuclei were labeled with DAPI (scale bars, 20 µm). **c** Analysis of cytotoxicity induced by DOX in cells transfected with hsiRNA Ctl (Ctl) or with two independent siMVP (MVP1, MVP2) and postincubated with AdCM for 72h using Incucyte Technology (expressed as the ratio of surviving cells post-incubated with AdCM over surviving NC cells). a.u: arbitrary units.
