# Supplementary material for: Adipocytes promote breast cancer resistance to chemotherapy, a process amplified by obesity: role of the major vault protein (MVP)
Source: Breast Cancer Res. 2019 Jan 17;21:7. doi: 10.1186/s13058-018-1088-6 (PMC6337862; doi:10.1186/s13058-018-1088-6)
Supplement: Supplementary file 8 — Figure S6. Hematoxylin/eosin staining of the tumor used to represent major vault protein (MVP) expression (Fig. 5e). (PDF 670 kb) [file 13058_2018_1088_MOESM8_ESM.pdf]

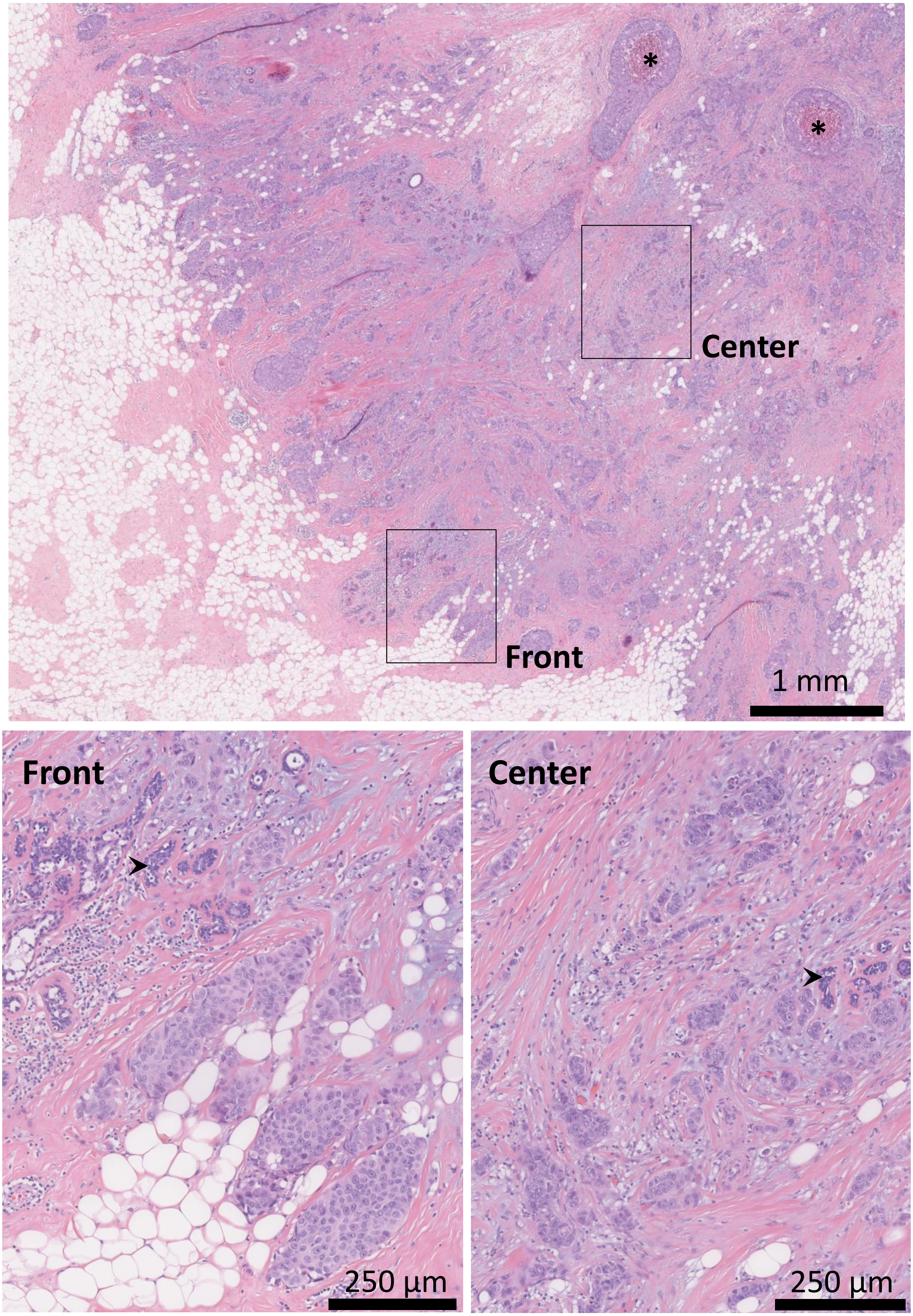

Figure S6. Hematoxylin/eosin staining of the tumor used to represent MVP expression (Fig. 5e). (Front) zoom of the invasive front in representative area; (Center) zoom of the center of the tumor in representative area. In situ carcinoma (black stars) and normal mammary glands (arrows heads) are considered as positive and negative internal controls respectively.

Lehuédé et al, Additional file 8
